# Shared and unique features of bacterial communities in native forest and vineyard phyllosphere

**DOI:** 10.1002/ece3.4949

**Published:** 2019-02-20

**Authors:** Toshiko Miura, Roland Sánchez, Luis E. Castañeda, Karina Godoy, Olga Barbosa

**Affiliations:** ^1^ Instituto de Ecología & Biodiversidad (IEB‐Chile) Santiago Chile; ^2^ Research Institute of Environment, Agriculture and Fisheries Osaka Japan; ^3^ Instituto de Ciencias Ambientales y Evolutivas, Facultad de Ciencias Universidad Austral de Chile Valdivia Chile; ^4^ Programa de Genética Humana, Instituto de Ciencias Biomédicas, Facultad de Medicina Universidad de Chile Santiago Chile

**Keywords:** biodiversity, conservation, forest, Mediterranean, sclerophyllous, viticulture

## Abstract

Phyllosphere bacteria have received little attention despite their important roles in shaping plant performance traits. In this study, we characterize the bacterial communities on leaves of native trees inhabiting sclerophyllous forests in central Chile, one of the world's biodiversity hotspots. Additionally, we provide profiles of bacterial communities on grape leaves and berries of organic and conventional vineyards. Results of 16S rRNA gene amplicon sequence analysis showed that 45% of OTUs were shared across forest leaves, grape leaves, and grape berries. Conventional management had higher number of OTUs shared with forest leaves than organic management. In addition, grape leaves subjected to conventional management had higher alpha diversity than those with organic management, while no significant effect of agricultural management was observed in grape berries. Indicator analysis showed that *Bdellovibrio*, *Beijerinckia*, and *Spirosoma* were typical for forest leaves, whereas *Enhydrobacter*, *Delftia*, *Proteiniclasticum*, *Arsenicicoccus*, and *Alkaliphilus* were typical for the vineyard phyllosphere. Regarding agricultural managements, *Beijerinckia*, *Sedimentibacter*, *Nesterenkonia*, *Gluconobacter*, *Conexibacter*, and *Anaeromyxobacter* were typical for conventional grape leaves, whereas no genus‐level indicator was found for organic vineyard leaves. These results provide new insights of the diversity patterns of the phyllosphere microbiome in native and cultivated lands and suggest that both of these microbiomes are connected and integrated systems.

## 1 INTRODUCTION

The aboveground parts of plants, so‐called phyllosphere, not only are among one of the most prevalent bacterial habitats on Earth, but also support diverse bacterial communities (Bringel & Couée, 2015; Yang, Crowley, Borneman, & Keen, 2001). Phyllosphere microorganisms provide specific ecosystem services and potentially mediate plant biodiversity–ecosystem function relationships (Kembel et al., 2014; Laforest‐Lapointe, Paquette, Messier, & Kembel, 2017; Vacher et al., 2016). For example, leaf‐associated bacteria have been shown to affect host growth (Saleem, Meckes, Pervaiz, & Traw, 2017) and protection against pathogen infection (Innerebner, Knief, & Vorholt, 2011). In addition, phyllosphere bacteria can influence the physicochemical properties of the environment, such as climate dynamics and the dynamics of numerous gaseous compounds of the surrounding atmosphere (Bringel & Couée, 2015). Therefore, phyllosphere bacterial communities may play substantial roles in key ecosystem processes that govern the global system. Nevertheless, as yet, the phyllosphere microbiome has received little attention compared to that of the rhizosphere and endosphere (Andreote, Gumiere, & Durrer, 2014).

Chilean Mediterranean ecosystems, located in central Chile, are major wine‐producing areas and are also one of the world's biodiversity hotspots where approximately 23% (2,500 species) of vascular plants are endemic (Arroyo, Cavieres, Marticorena, & Muñoz‐Schick, 1995; Myers, Mittermeier, Mittermeier, Fonseca, & Kent, 2000). Land use change mediated by vineyard expansion poses a threat to ecosystem services provided by native habitats (Barbosa & Villagra, 2015; Viers et al., 2013). Currently, in the Chilean wine industry, on‐farm and landscape‐scale management techniques have been taken into consideration to reduce the negative effects of vineyard expansion. Specifically, the conservation of sclerophyll vegetation and incorporation in the vineyard matrix have been encouraged (natural patches or biological corridors; Viers et al., 2013). However, the bacterial diversity of the phyllosphere in Chilean sclerophyllous forests and the role that these bacteria play in Chilean Mediterranean ecosystems are completely unknown. This may prevent us from formulating adequate land‐use strategies for the conservation of biodiversity, sustainable viticulture, and the development of biotechnological solutions for the wine industry. It is also important to the wine industry to understand how agricultural management affects the microbial community of grapevines because grapevine microorganisms can affect the production of healthy grapes (Barata, Malfeito‐Ferreira, & Loureiro, 2012; Pinto & Gomes, 2016) and wine quality (Bokulich et al., 2016; Knight, Klaere, Fedrizzi, & Goddard, 2015; Mezzasalma et al., 2017). The natural phyllosphere bacteria of the grapevine are likely to be highly resilient to agricultural treatments; studies have shown that differences in management, including chemical fungicides (Perazzolli et al., 2014), biological control (Perazzolli et al., 2014), and conventional, organic and biodynamic systems (Kecskeméti, Berkelmann‐Löhnertz, & Reineke, 2016), do not affect bacterial diversity indices. On the other hand, the composition of bacterial communities found on wine grapes has been shown to be affected by agricultural practices, as differences in dominant taxonomic groups between organic and conventionally managed grapes have been observed (Pinto & Gomes, 2016).

In a previous paper, we analyzed microbial community composition (both bacteria and fungi) on leaves and grapes among vineyards and correlated them with geographical distance (Miura, Sánchez, Castañeda, Godoy, & Barbosa, 2017). We found that while bacterial community dissimilarity was not correlated with geographic distance, fungal community dissimilarities in both the leaf and berries increased with geographic distance. Those results suggest the important role spatial processes play in structuring the communities at local scales. This article builds on those results and aims at evaluating what factors might be affecting the structure and composition of bacteria in these human‐modified landscapes. In particular, we determined whether bacterial diversity differs among Carmenere vineyards subjected to different agricultural management practices and the surrounding native forests. To that end, we sampled bacterial diversity and community composition of leaves from sclerophyllous forests adjacent to vineyards and used the previously reported vineyard bacterial diversity data from Miura et al. (2017).

## 2 MATERIALS AND METHODS

### 2.1 Sampling

**Figure 1:**
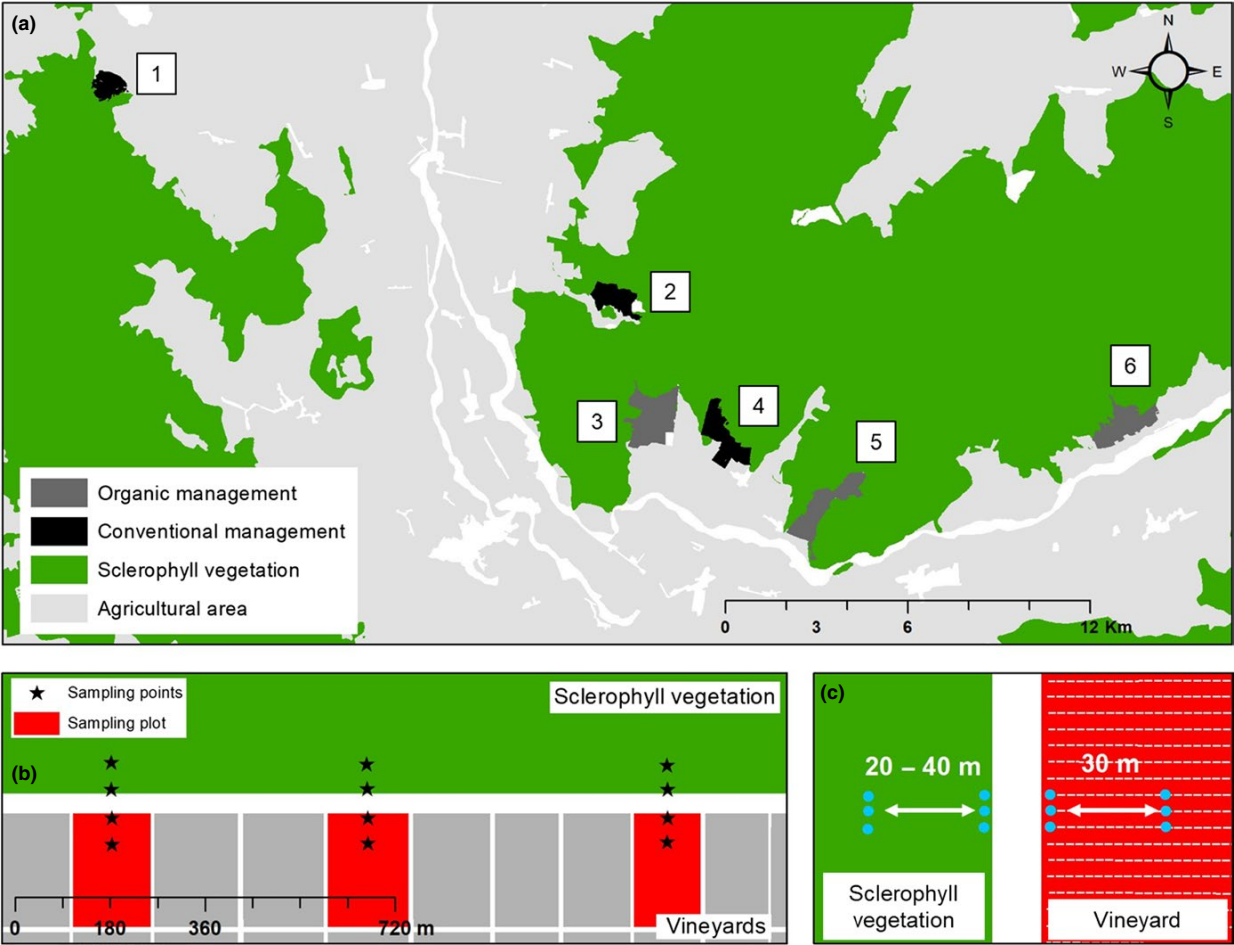
Geographical location and sampling design of the six sampling sites in Valle de Colchagua, Chile (a), and sampling design at each site (b,c)

Sampling methodology for leaves and berries from vineyards is described in Miura et al. (2017). In brief, samples were collected from six vineyards (three with conventional management and three with organic management) and from their surrounding sclerophyllous forests (Figure 1a). All vineyards and forests were located in the Colchagua Valley, Chile (34°15′S–34°50′S: 70°15′W–72°00′W). Samples of grape leaves and berries were taken during the last week before the Carmenere harvest of 2014 (April in the Southern Hemisphere), and sampling covered approximately 35 km. The three vineyards with organic farming practices were characterized by the use of organic fertilizers and biological control to manage pests, whereas the three vineyards with conventional farming practices were characterized by the use of inorganic fertilizer and synthetic pesticides and herbicides to control pests and weeds. *Bacilus subtilus* and *Bacillus thuringiensis kurstaki* were applied as a biological fungicide and a biological insecticide, respectively, in the organic vineyards in October or November.

At each vineyard, grape leaves and bunches were taken from three plots of the Carmenere cultivar adjacent to the sclerophyllous forest (Figure 1b). Two sampling points within a plot were selected: One sampling point was located close to the forest and the other sampling point was located 30 m toward the edge of the vineyard (Figure 1b). In each sampling point, a total of three undamaged grape bunches and 10 g of leaves were collected from three grapevines (Figure 1c). In total, six grape bunches and 20 g of leaves (approximately 18 leaves) were collected from each plot. Leaves and bunches were placed into separate sterile plastic bags.

In each forest, leaves were collected from three sites adjacent to the vineyard (Figure 1b). Two sampling points within a site were selected: One sampling point was located close to the vineyard and the other sampling point was located 20–40 m toward the edge of the forest (Figure 1b). From each sampling point, total 10 g of leaves was taken from four different trees (Figure 1c). In total, 20 g of leaves (88 ± 65 leaves) was collected from each site. If more than one tree species was located at a given sampling point, all of the trees were sampled equally to generate a 20‐g composite sample. All of the forests sampled included native tree species commonly found in Chilean sclerophyllous forests, including litre (*Lithraea caustica*), boldo (*Peumus boldus*), peumo (*Cryptocarya alba*), quillay (*Quillaja saponaria*), and espino (*Acacia caven*) (for more detail of species composition in each sample, please see Supporting Information Table S1). We took leaves at a height of 1–1.5 m. Fruits from forest trees were almost absent during the autumn, and thus, they were not included in the sampling. All of the samples were collected using surgical gloves and sterilized scissors. Samples were then stored in hermetic storage bags sterilized with ethylene oxide and maintained in dry ice until arrival at the laboratory at the Universidad Austral de Chile (Valdivia, Chile). At the laboratory, samples were stored at −80ºC until DNA extraction was performed. There were 18 samples (six sites and three plots in each site) for each forest leaf, grape leaf, and grape berry sample. In total, 54 samples were collected. For grape berry samples, a total of 42 berries were picked from six grape bunches of each plot.

### 2.2 DNA extraction and Illumina sequencing

Each sample was suspended in 200 ml of a 0.9% NaCl–0.02% Tween‐20 solution and shaken for 2 hr at 100 rpm at room temperature using a Multi‐rotator RS‐60 (BioSan). The wash solution was filtered using sterilized gauze to eliminate large pieces of plant tissue. This was followed by centrifugation for 5 min at 230 *g* to eliminate small quantities of plant residue. The supernatant was transferred to 50‐ml tubes and centrifuged for 20 min at 5,660 *g*. Genomic DNA was extracted from the resulting pellets using a PowerSoil DNA isolation kit (MoBio) following the manufacturer's instructions. After extraction, DNA was quantified by fluorescence with a Quan‐iT PicoGreen dsDNA kit (Invitrogen).

To characterize bacterial diversity, PCR amplicon sequencing was performed. A pair of primers recognizing the V5–V6 region of the 16S rRNA gene was used: 799f (AACMGGATTAGATACCCKG) and 1115r (AGGGTTGCGCTCGTTG) (Redford, Bowers, Knight, Linhart, & Fierer, 2010). These primers do not amplify chloroplast nor cyanobacterial DNA. The first PCR was for amplification (25 cycles), and the second PCR was to add the identification to the samples (eight cycles). For both PCRs, KAPA HiFi HotStart ReadyMix (KAPA Biosystems) was used to amplify the target DNA region, and AMPure XP beads (Beckman Coulter) were used to purify the amplicons. PCR conditions used were as follows: 95°C for 3 min, 25 or eight cycles of 95°C for 30 s, 55°C for 30 s, 72°C for 30 s, and 72°C for 5 min. Amplicon sequencing was performed using 250‐bp paired‐end sequencing on an Illumina MiSeq sequencer (Illumina) following the 16S Metagenomic Sequencing Library Preparation protocol in Australomics laboratory of the Universidad Austral de Chile. The sequence data are available in GenBank under BioProject number PRJNA392467.

### 2.3 Data analysis

Raw sequences were quality filtered for a q‐value higher than 26 and for sequences longer than 150 bp (Bálint, Schmidt, Sharma, Thines, & Schmitt, 2014). Forward and reverse filtered sequences were paired using PANDAseq with a minimum read overlap between forward and reverse sequences of 5 bp (Masella, Bartram, Truszkowski, Brown, & Neufeld, 2012). The raw sequences were analyzed using QIIME v1.9.1 (Caporaso et al., 2010). The open‐reference OTU‐picking strategy was used to generate operational taxonomic units (OTUs; 97% similarity). The usearch 6.1 algorithm was used to cluster OTUs, and uclust was used to assign taxon identifications (Edgar, 2010) by blasting against the Greengenes 16S rRNA gene 13_8 database and employing 97% pairwise identities (McDonald et al., 2012). The usearch script also includes the removal of chimera sequences. Also, nonbacterial sequences (i.e., archaeal, eukaryotic, chloroplast, or mitochondrial) were removed with the filter_taxa_from_otu_table.py script, and any OTUs comprising less than 0.001% of the total sequences were removed prior to further analysis (Bokulich et al., 2013). A phylogenetic tree comprising all OTUs was generated using FastTree (Price, Dehal, Arkin, Rojas, & Brodie, 2010). One leaf sample taken from a vineyard was discarded during the analysis because of low sequence counts (only 30 sequences). Therefore, a total of 53 samples were used for statistical analysis, of which 35 samples were previously reported in Miura et al. (2017) and 18 samples were obtained in this study from the sampling of the native forest.

### 2.4 Statistical analysis

We standardized the OTU table to generate measures of alpha diversity and community composition for each sample by normalizing with the cumulative sum scaling (CSS) (Paulson, Stine, Bravo, & Pop, 2013) implemented in QIIME. The alpha diversity in each habitat was compared using multiple metrics for observed OTU richness, phylogenetic diversity (PD, Faith, 1992), the Shannon diversity index (*H* = ‐sum(p_i x ln(p_i)), where p_i is the proportional abundance of OTUs), and the Pielou's evenness index (*J* = −*H*/log(*S*), where *H* is the Shannon diversity index and *S* is the observed OTU richness) calculated in R. The alpha diversity data were tested for normality using Shapiro–Wilk tests; no violations of normality were detected. To measure spatial beta diversity, we used the *betadisper* function in the vegan package (Oksanen et al., 2014). This function performs a principal coordinate analysis of species compositions for a group of communities and for each returns a distance from the group centroid as a measure of multivariate community dispersion. Taxonomic dissimilarity was calculated based on Bray–Curtis distance, and phylogenetic dissimilarity was calculated based on UniFrac distance. Weighted and unweighted UniFrac distances among samples were calculated in QIIME. A one‐way analysis of variance (ANOVA) was used to assess the effect of habitat (forest leaf, grape leaf, grape berry) and the effect of agricultural management (organic and conventional) within grape leaves or berries on alpha diversity and beta diversity. We performed Tukey's multiple comparisons to compare the diversity indices between the different habitats. Principal coordinate analysis (PCoA) was applied to visualize the samples based on abundance and binary (presence–absence) using Bray–Curtis and UniFrac distance metrics. The effect of habitat (forest leaf, grape leaf, grape berry) and management within grapevine organs on these distance matrices were analyzed by permutational multivariate analyses of variance (PERMANOVA; Anderson, 2001). These analyses were conducted using the Adonis function of the R vegan package with 999 permutations. When the PERMANOVA suggested significant differences in community structure between groups, we then determined which taxa were driving those differences by identifying indicator species using the R package *indicspecies* (Cáceres & Legendre, 2009). This analysis calculates an indicator value (IndVal) that measures the association between OTUs with each group or combination of groups and then identifies the group corresponding to the highest association value. We defined indicator OTUs based on an IndVal of >0.70 and a *p*‐value <0.05 assessed after 999 permutation tests. Genus‐level indicators were also identified and visualized using the R package *RAM* (Chen, Simpson, & Levesque, 2016) employing the same parameters as the OTU‐level indicator identification.

## 3 RESULTS

From the 53 samples, a total of 1,712,370 reads were generated. After omitting 17% of low‐quality sequences, a total of 1,421,255 reads were generated, and the average number of reads per sample was 26,319 ± 16,575 (mean ± *SD*) bp (see Supporting Information Table S2). Clustering at 97% identity produced 4,882 OTUs across all samples. Rarefaction plots for observed OTUs were close to reaching a plateau for most samples (see Supporting Information Figure S1), indicating good overall OTU coverage afforded by deep sequencing.

### 3.1 Diversity of phyllosphere bacterial communities

**Figure 2:**
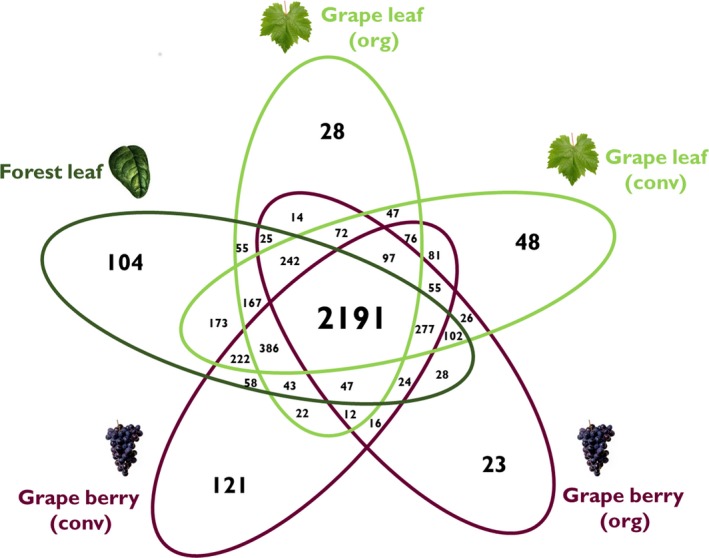
Venn diagram showing the number of shared or unique OTUs among bacterial communities of sclerophyllous and vineyard vegetation

The Venn diagram showed that 45% of all OTUs were shared among samples, and 6.6% (324 OTUs) of the all OTUs were unique to the given tissue–management combination (Figure 2). Interestingly, some malolactic bacteria including *Lactobacillus* and *Leuconostoc* were among the shared OTUs (see Supporting Information Table S2).

**Figure 3:**
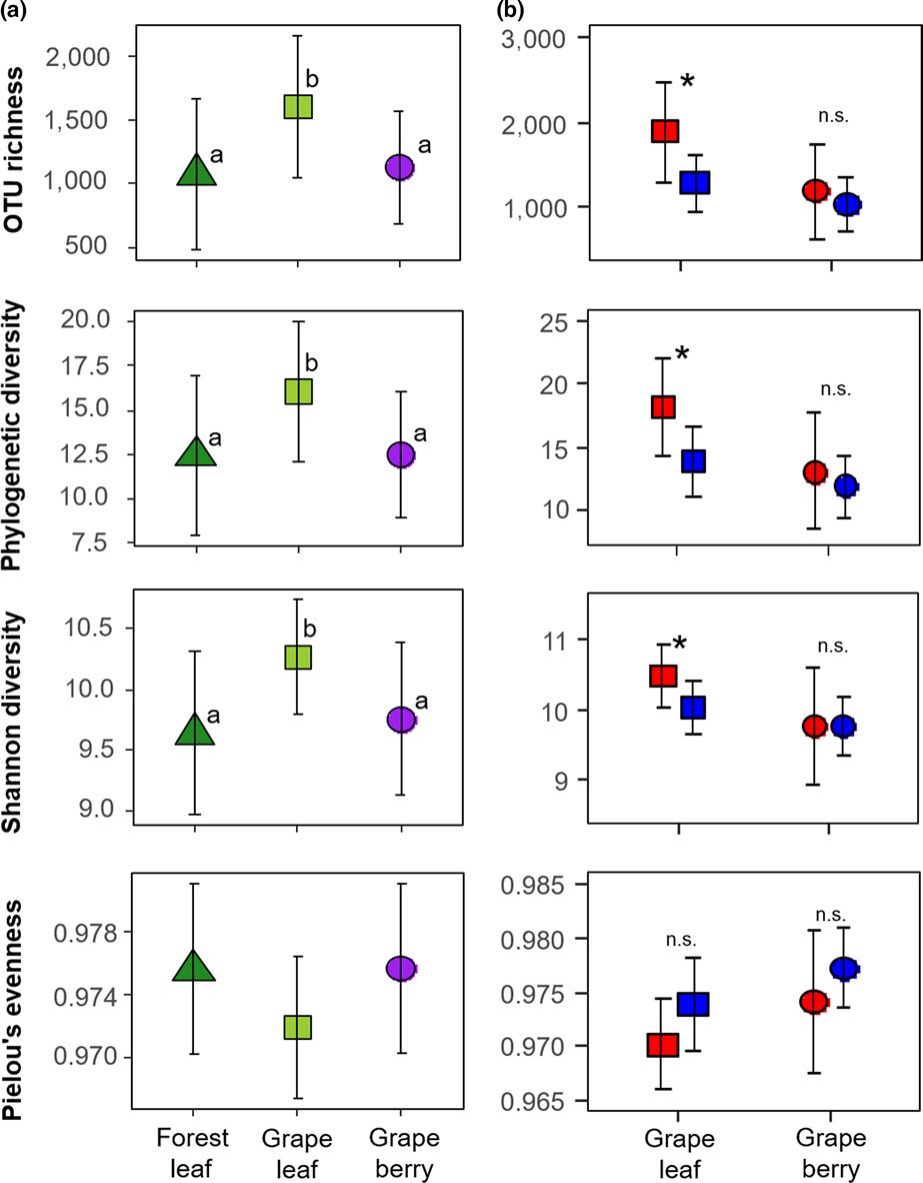
Variation in alpha diversity of the bacterial communities on the forest leaf, grape leaf, and grape berry (a) and conventional (red color) and organic management (blue color) (b). The error bars represent standard deviations of means. Different letters indicate a significant difference between the three habitats according to Tukey's multiple comparisons (*p* < 0.05). The asterisk indicates a significant difference between the two management types within grapevine organs determined by an ANOVA (*p* < 0.05)

The ANOVA indicated that there was a significant effect of habitat on OTU richness (*F*_2,50_ = 5.28, *p < *0.01), phylogenetic diversity (PD; *F*_2,50_ = 4.60, *p < *0.05), and Shannon diversity (*F*_2,50_ = 5.30, *p < *0.01), whereas the effect on Pielou's evenness was not significant (*F*_2,50_ = 3.0, *p = *0.06). Grape leaves had the highest mean values of OTU richness, PD, and Shannon diversity among these habitats. Tukey's post hoc multiple comparisons showed that differences in these variables between grape leaves and the other two habitats were statistically significant (Figure 3a).

There was a significant effect of agricultural management on the OTU richness (*F*_1,15_ = 6.60, *p* < 0.05), the PD (*F*_1,15_ = 6.52, *p < *0.05), and the Shannon diversity (*F*_1,15_ = 5.33, *p < *0.05) of grape leaves (Figure 3b). On the other hand, these indices for grape berries were less affected by agricultural management (*F*_1,16_ = 0.50, *p = *0.49; *F*_1,16_ = 0.49, *p = *0.49; *F*_1,16_ = 0.003, *p = *0.96, respectively). Pielou's evenness of grape leaves (*F*_1,15_ = 3.46, *p = *0.08) and berries (*F*_1,16_ = 1.60, *p = *0.23) were not significantly affected by agricultural management.

**Figure 4:**
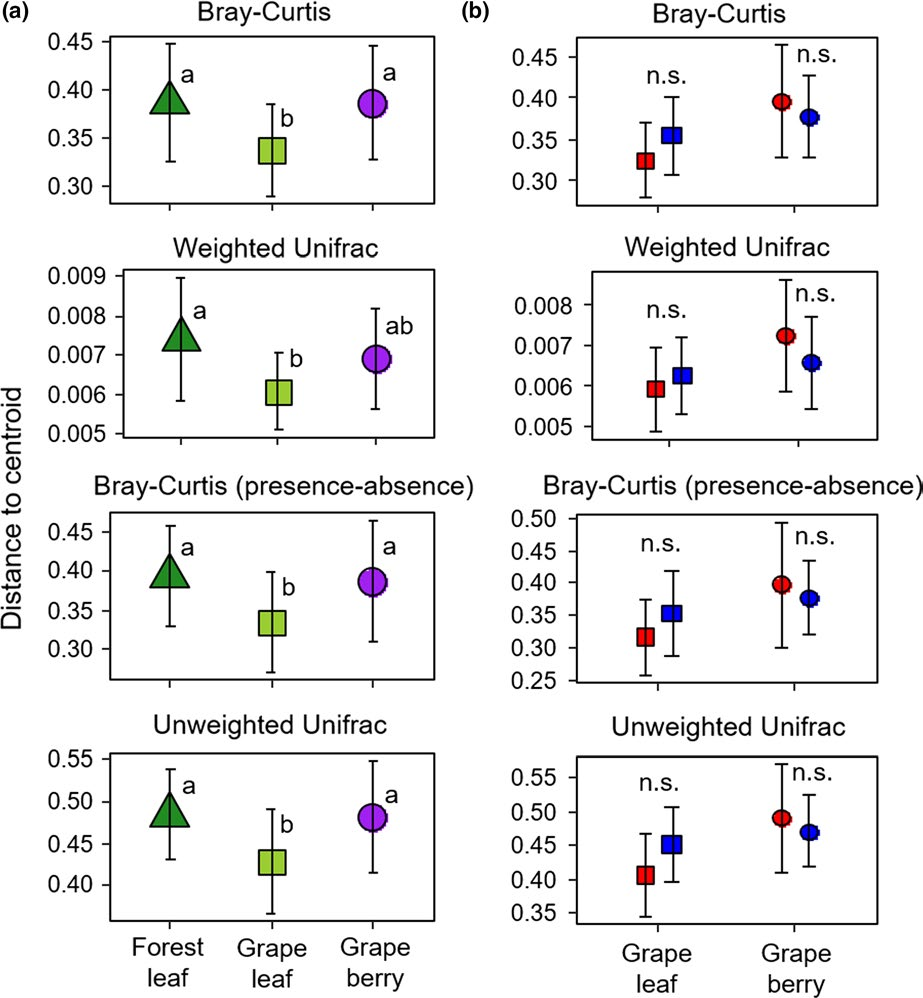
Variation in beta diversity (average distance to group centroid) of the bacterial communities on the forest leaf, grape leaf, and grape berry (a) and conventional (red color) and organic management (blue color) (b). The error bars represent standard deviations of means. Different letters indicate a significant difference between the three habitats according to Tukey's multiple comparisons (*p* < 0.05). The asterisk indicates a significant difference between the two management types within grapevine organs determined by an ANOVA (*p* < 0.05)

Beta diversity showed significant differences in the distance to the centroid between among habitat (Bray–Curtis: *F*_2,50_ = 4.41, *p < *0.05; weighted UniFrac: *F*_2,50_ = 4.64, *p < *0.05; Bray–Curtis presence–absence: *F*_2,50_ = 4.00, *p < *0.05; unweighted UniFrac: *F*_2,50_ = 4.76, *p < *0.05). Grape leaves had the lowest mean values of beta diversity among these habitats. Tukey's post hoc multiple comparisons showed that differences in these variables between grape leaves and the other two habitats were statistically significant (Figure 4a). There was no significant effect of agricultural management on the beta diversity (Figure 4b).

### 3.2 Community composition of phyllosphere bacteria

**Figure 5:**
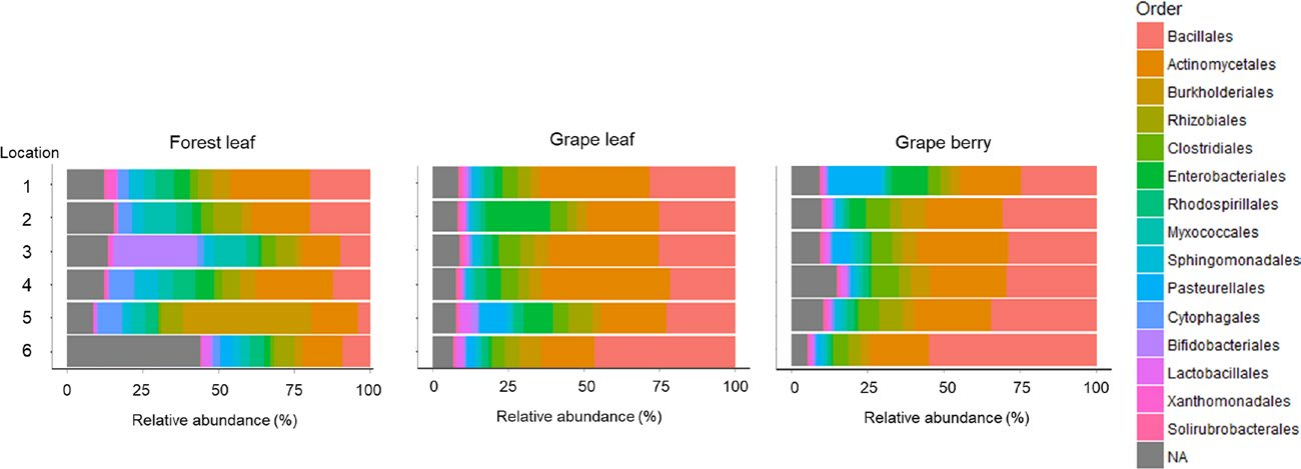
Taxonomic composition (order level) of bacterial communities in sclerophyllous tree leaves, grape leaves, and grape berries. OTU tables representing sequence counts were used for making this taxonomy summary

**Figure 6:**
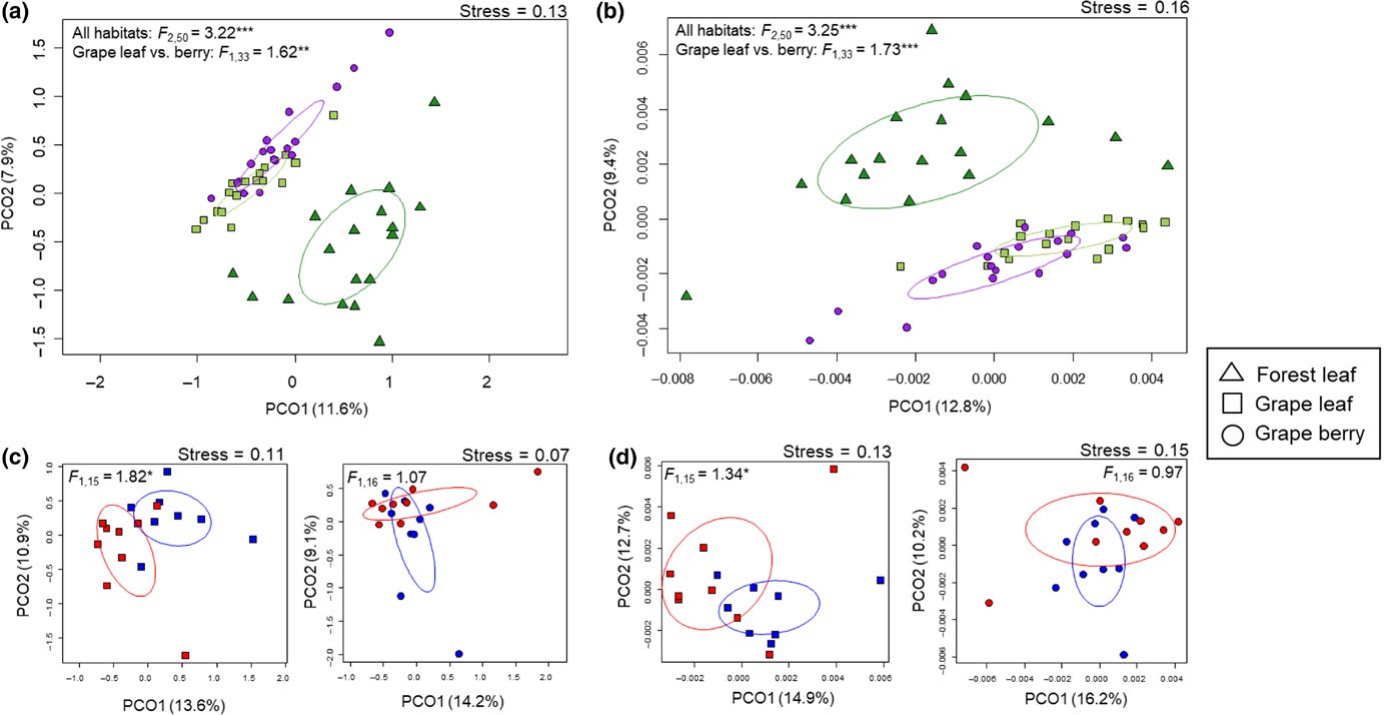
Principal coordinate analysis (PCoA) of bacterial communities for forest leaves, grape leaves, and berries based on Bray–Curtis (a) and weighted UniFrac distance (b). PCoA of bacterial communities for grape leaves and berries between conventional and organic vineyards (conventional in red and organic in blue) based on Bray–Curtis (c) and weighted UniFrac distance (d). Results of the PERMANOVAs conducted for habitat or for agricultural management are shown. The asterisks indicate statistical significance: *p < 0.05, **p < 0.01, ***p < 0.001

**Figure 7:**
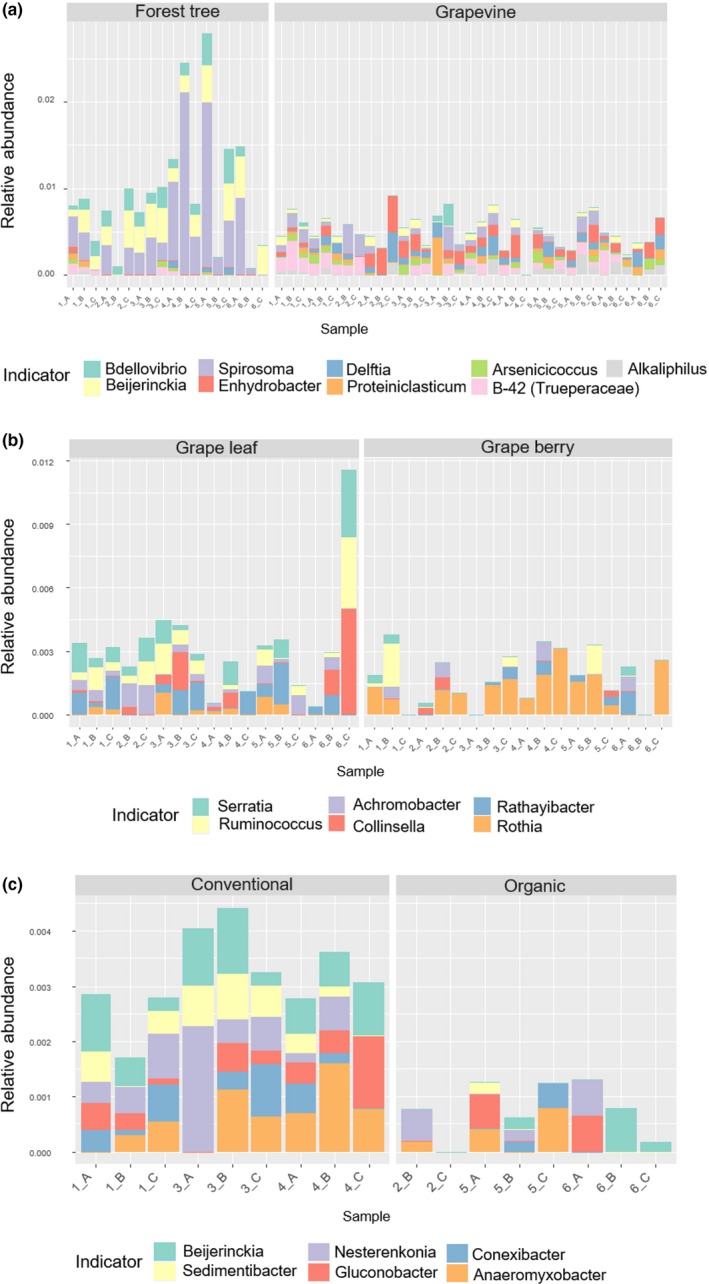
The relative abundance of indicator genera for forest and grapevines (a), grape leaf and grape berry (b), and conventional and organic grape leaves (c). Cumulative sum scaling normalized OTU tables were used for making the figure

The most abundantly represented orders in forest leaves were *Bacillales*, *Actinomycetales,* and *Burkholderiales* (Figure 5). The PCoA plot showed that forest leaves were clearly separated from grape leaves and berries, and the PERMANOVA results revealed that the taxonomic (Bray–Curtis) and phylogenetic (UniFrac) OTU compositions among habitats were significantly different (Figure 6a,b). Those results were similar for presence/absence data (see Supporting Information Figure S2). Indicator analyses revealed phylogenetic variability of indicators that contributed to the dissimilarity between the bacterial community composition on forest leaves and vineyard grapes (Figure 7a). Forest leaves had 72 indicator OTUs, whereas the vineyard phyllosphere (grape leaves and berries) had 14 indicator OTUs (see Supporting Information Table S4). At the genus level, *Bdellovibrio*, *Beijerinckia*, and *Spirosoma* were typical for forest leaves, whereas *Enhydrobacter*, *Delftia*, *Proteiniclasticum*, *Arsenicicoccus*, *Alkaliphilus*, and *B‐42* (*Trueperaceae*) were typical for the vineyard phyllosphere (Figure 7a).

The most abundantly represented orders in grape leaves as well as in grape berries were *Bacillales* and *Actinomycetales* (Figure 5). According to the PERMANOVA, there was a significant difference in OTU composition between grape leaves and berries (Figure 6a,b). The indicator analysis showed that grape leaves had 38 indicator OTUs, whereas no indicator OTUs was found for grape berries (see Supporting Information Table S5). On the other hand, the indicator analysis at the genus level showed that members of the genera *Serratia*, *Ruminococcus*, *Achromobacter*, *Collinsella,* and *Rathayibacter* were typical for grape leaves, whereas *Rothia* were typical for grape berries (Figure 7b).

The PERMANOVA testing for the effects of agricultural management on grape bacterial community composition revealed that bacterial communities in grape leaves were significantly different between organic and conventional management, but the effect was not significant in grape berries (Figure 6c,d). Leaves from conventional vineyards had 186 indicator OTUs, whereas leaves from organic vineyards had two indicator OTUs (see Supporting Information Table S6). *Beijerinckia*, *Sedimentibacter*, *Nesterenkonia*, *Gluconobacter*, *Conexibacter,* and *Anaeromyxobacter* were typical for conventional grape leaves, whereas no genus‐level indicator was found for organic vineyard leaves (Figure 7c).

## 4 DISCUSSION

### 4.1 Phyllosphere of sclerophyllous vegetations and vineyards

This study describes the bacteria community structure inhabiting the phyllosphere of grapevines and adjacent native forest trees. The results of our analyses indicated that approximately half of all OTUs were shared by these two habitat types (Figure 2). Interestingly, among the OTUs shared across habitats were some lactic acid bacteria that have important functions in the malolactic fermentation of wine. The presence of lactic acid bacteria has also been reported in forest and vineyard soils in other vineyards in the same valley sampled here (Castañeda & Barbosa, 2017). “Where do grape‐associated microorganisms come from?” is an interesting and important question for the wine industry in the context of microbial *terroir *(Gilbert, Lelie, & Zarraonaindia, 2014). However, almost no other studies have evaluated the contribution of the surrounding landscape to the vineyard microbiome. Recently, it has been suggested that native forests near vineyards are significant sources of fungal communities in harvested juice and ferments (Morrison‐Whittle & Goddard, 2017). In addition, Fort, Robin, Capdevielle, Delière, and Vacher (2016) reports that airborne fungal community composition does not differ significantly between forest patches and adjacent vineyards, which suggests that the dispersal of foliar fungal communities is not limited at landscape scales. Regarding bacteria in grape leaves and berries, our previous study has indicated that they are less sensitive to spatial effects than are fungi (Miura et al., 2017). This might be one possible reason why many bacterial species were shared between different habitats given that vineyard plots in this study were very near to the forest (Figure 1).

Host taxonomic identity is an important driver of phyllosphere bacterial community structure (Kembel et al., 2014; Redford et al., 2010; Whipps, Hand, Pink, & Bending, 2008). Previous studies have shown that the bacterial community structure found on leaves is very similar between individuals of the same plant species but varies significantly between different plant species (Lambais, Crowley, Cury, Büll, & Rodrigues, 2006; Yang et al., 2001). Our results showed that forest leaves had more heterogeneity (higher beta diversity) of bacterial communities among sites than grape leaves. This is reasonable because the composition of plant species contained in the forest sample varies from sample to sample. We also expected that the phyllosphere of multiple plant species (e.g., forest phyllosphere) would have more diverse bacterial communities than the phyllosphere of a single plant species (e.g., vineyard phyllosphere); however, the OTU richness and diversity found in forest leaves were lower than that found in grape leaves. One possible explanation is that sclerophyllous leaves have hard and thick cuticles. The cuticle functions as a barrier for invasive microorganisms (Bringel & Couée, 2015; Yeats & Rose, 2013). In some cases, cuticle thickness has been correlated with resistance to pathogen diseases (Martin, 1964; Yang, Verma, & Lees, 1992). Due to the presence of hard and thick cuticles, sclerophyllous leaves may restrict the invasion of bacterial species more than grape leaves. Furthermore, differences in the composition of bacterial communities between forests and vineyards may also be related to differences in alpha diversity. The members of the genus *Bdellovibrio*, which was the indicator genus of forest leaves, are known as obligately predatorial bacteria, which prey on other gram‐negative bacteria (Lambert, Morehouse, Chang, & Sockett, 2006). Feng et al. (2017) have reported that *Bdellovibrio* predation significantly alters the species composition in activated sludges, with the relative abundance of >90% of the community being reduced by predation. Savka, Dessaux, Oger, and Rossbach (2002) also reviewed *Bdellovibrio *as predation‐based biocontrol agents against plant pathogenic bacteria.

### 4.2 Conventional versus organic agricultural management in vineyards

We showed that conventional management had higher number of OTUs shared with forest leaves than organic management for both grape leaves and berries. In addition, it was observed that grape leaves subjected to conventional management had higher bacterial OTU richness and diversity than those subjected to organic management. Organic management involves the use of microorganisms as biological control. In the vineyards of this study, substantial amounts of *Bacillus subtilis *were applied as biocontrol against grapevine fungal diseases. This could be a factor that affected bacterial diversity, as the main biocontrol mechanism of this species is competition for nutrients and space with other microorganisms (Raupach & Kloepper, 1998; Romero et al., 2007). Despite this, surprisingly, we did not find this species nor *B. thuringiensis kurstaki* as indicator OTUs in organic grape leaves. Nevertheless, Wei, Hu, and Xu (2016) has shown that the amount of introduced *B. subtilis* declines rapidly after several days of introduction and does not greatly affect the fungal and bacterial communities of strawberry leaves. On the other hand, another study has shown that *B. thuringiensis* treatments alter the microbial community composition of pepper phyllospheres even though this species was not dominant within the phyllosphere following its application to the plant surface (Zhang et al., 2008). Further studies are needed to determine what specific components of agricultural practices affect bacterial communities.

In contrast to grape leaves, grape berries were less affected by agricultural management. This result supports previous studies indicating that the natural phyllosphere bacteria of the grape berry are likely to be highly resilient to agricultural treatment (Kecskeméti et al., 2016). Additionally, our study suggests that the sensitivity to agricultural management differs between leaf‐associated and berry‐associated bacterial communities. However, our sampling was conducted only at one time of the year and is thus insufficient to properly characterize the dynamics of phyllosphere bacterial composition.

## 5 CONCLUSION

This study focused on the phyllosphere microbiome of Chilean Mediterranean ecosystems, which until now had not been studied in depth. This is the first study that evaluates microbial diversity and community structure in Chilean vineyards in a landscape context. Our results show the importance of evaluating agricultural plots and their associated microbial diversity in a landscape context given the recent evidence that strongly suggests that both of these vineyards and adjacent native vegetations are connected and integrated systems. This can provide important insights to advance studies focused on dealing with the mechanisms involved in structuring communities and microbial diversity. Overall, the results generated here could guide appropriate landscape and agricultural management to maximize ecosystem services and minimize ecosystem disservices provided by microorganisms.

## CONFLICT OF INTEREST

The authors declare no conflict of interest.

## AUTHOR CONTRIBUTIONS

Toshiko Miura analyzed the data, wrote the manuscript, prepared the figures and tables, and reviewed drafts of the manuscript. Roland Sánchez conceived and designed the experiments, analyzed the data, performed the experiments, and reviewed drafts of the manuscript. Luis E. Castañeda conceived and designed the experiments and reviewed drafts of the manuscript. Karina Godoy performed the experiments and reviewed drafts of the manuscript. Olga Barbosa conceived and designed the experiments, contributed reagents/materials/analysis tools, and reviewed drafts of the manuscript.

## DATA ACCESSIBILITY

The sequence data are available in GenBank under BioProject number PRJNA392467.
