# Supplementary material for: Shared and unique features of bacterial communities in native forest and vineyard phyllosphere
Source: Ecol Evol. 2019 Feb 20;9(6):3295–305. doi: 10.1002/ece3.4949 (PMC6434556; doi:10.1002/ece3.4949)
Supplement: Supplementary file 1 [file ECE3-9-3295-s001.docx]

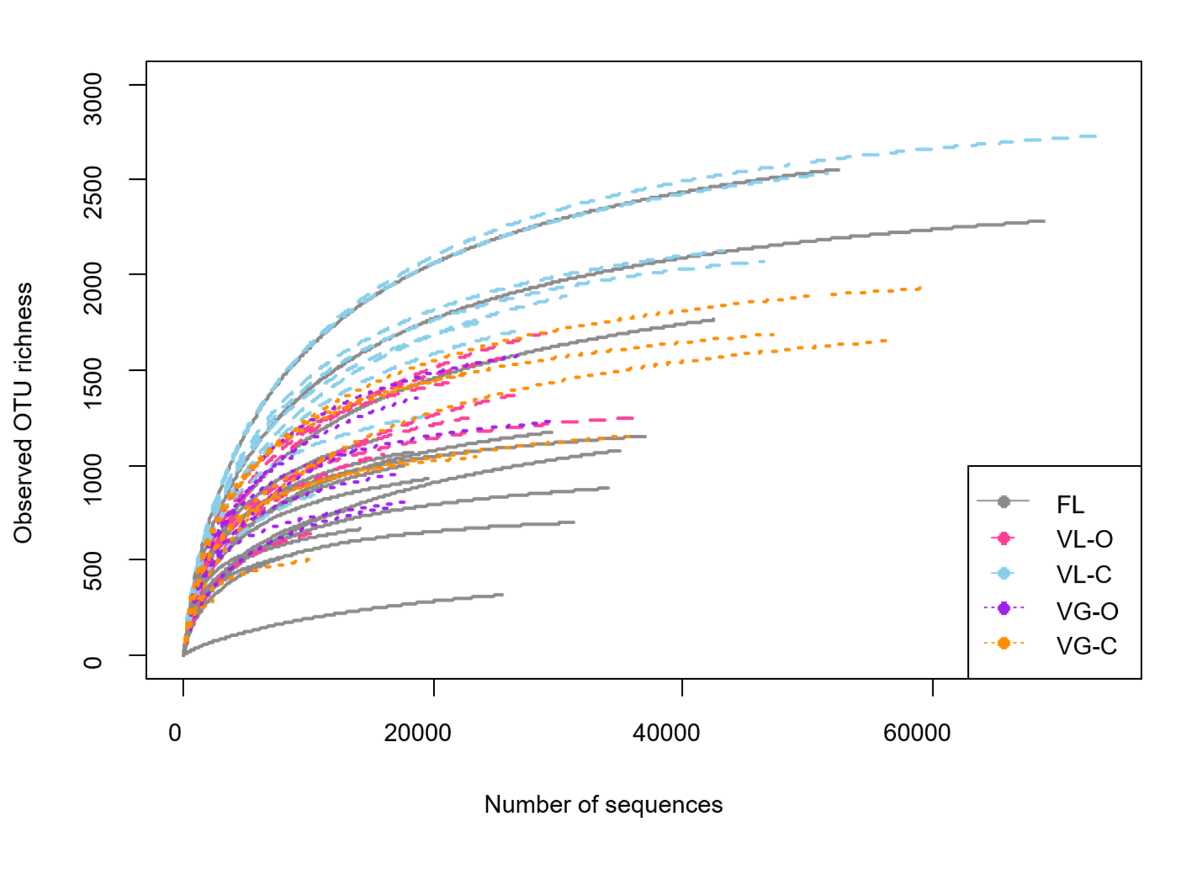


**Figure S1** Rarefaction curves of the observed bacterial OTU richness at 97% sequence similarity. FL, forest leaf; VL-O, grape leaf (organic); VL-C, grape leaf (conventional); VG-O, grape berry (organic); VG-C, grape berry (conventional).
