# Supplementary material for: Shared and unique features of bacterial communities in native forest and vineyard phyllosphere
Source: Ecol Evol. 2019 Feb 20;9(6):3295–305. doi: 10.1002/ece3.4949 (PMC6434556; doi:10.1002/ece3.4949)
Supplement: Supplementary file 2 [file ECE3-9-3295-s002.docx]

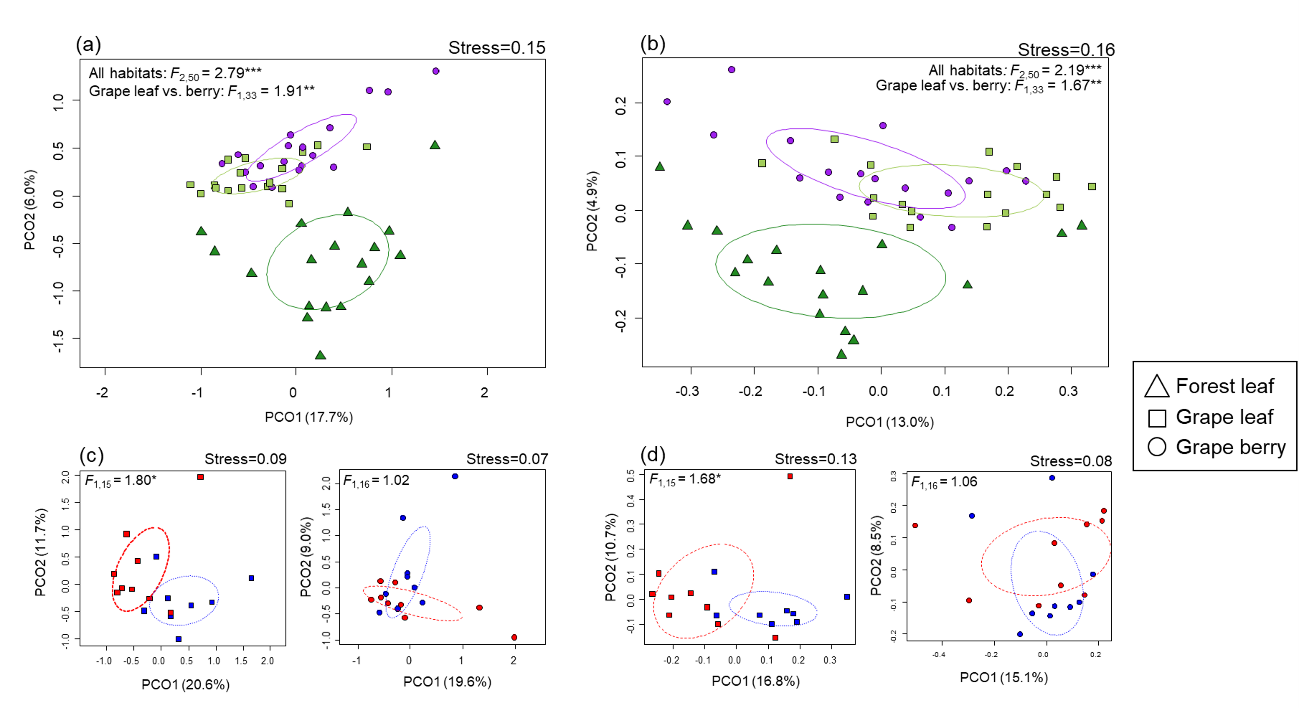


**Figure S2.** PCoA of bacterial communities for forest leaves, grape leaves and berries based on Bray-Curtis (presence-absence data) **(a)** and unweighted UniFrac distance **(b)**. PCoA of bacterial communities for grape leaves and berries between conventional and organic vineyards (conventional in red and organic in blue) based on Bray-Curtis (presence-absence data) **(c)** and unweighted UniFrac distance **(d)**. Results of the PERMANOVAs conducted for habitat or for agricultural management are shown. The asterisks indicate statistical significance: **P* < 0.05, ***P* < 0.01, ****P* < 0.001.
