# Supplementary material for: Shared and unique features of bacterial communities in native forest and vineyard phyllosphere
Source: Ecol Evol. 2019 Feb 20;9(6):3295–305. doi: 10.1002/ece3.4949 (PMC6434556; doi:10.1002/ece3.4949)
Supplement: Supplementary file 4 [file ECE3-9-3295-s004.docx]

**Supporting information**

**Table S2** Sequence counts after quality checks and low nearest sequenced taxon index (NSTI) values per sample

Vineyard #2 leaf sample taken from plot A was discarded in analysis because of low sequence counts (only 30 sequences).
