# Supplementary material for: Shared and unique features of bacterial communities in native forest and vineyard phyllosphere
Source: Ecol Evol. 2019 Feb 20;9(6):3295–305. doi: 10.1002/ece3.4949 (PMC6434556; doi:10.1002/ece3.4949)
Supplement: Supplementary file 5 [file ECE3-9-3295-s005.docx]

**Table S2** Relative abundance (% of total sequence) of malolactic bacteria in forest and vineyard habitats.

Values represent means. n.d., not detected.
