# Supplementary material for: Shared and unique features of bacterial communities in native forest and vineyard phyllosphere
Source: Ecol Evol. 2019 Feb 20;9(6):3295–305. doi: 10.1002/ece3.4949 (PMC6434556; doi:10.1002/ece3.4949)
Supplement: Supplementary file 6 [file ECE3-9-3295-s006.docx]

Supplementary Table S3. Indicator OTUs of forest vs. vineyard

| OTU ID | group | A | B | stat | p.value | Taxonomy |
| --- | --- | --- | --- | --- | --- | --- |
| 957772 | forest | 0.9141 | 0.9444 | 0.929 | 0.001 | k__Bacteria;p__Proteobacteria;c__Deltaproteobacteria;o__Myxococcales;f__Cystobacterineae;g__;s__ |
| New.ReferenceOTU4417 | forest | 0.8557 | 1 | 0.925 | 0.001 | k__Bacteria;p__Proteobacteria;c__Alphaproteobacteria;o__Rhizobiales;f__Methylocystaceae;g__;s__ |
| New.ReferenceOTU2 | forest | 0.8538 | 1 | 0.924 | 0.001 | Unassigned;NA;NA;NA;NA;NA;NA |
| 137768 | forest | 0.8875 | 0.9444 | 0.916 | 0.001 | k__Bacteria;p__Proteobacteria;c__Deltaproteobacteria;o__Myxococcales;f__Cystobacterineae;g__;s__ |
| New.ReferenceOTU6 | forest | 0.8333 | 1 | 0.913 | 0.001 | k__Bacteria;p__Proteobacteria;c__Alphaproteobacteria;o__Rhodospirillales;f__Acetobacteraceae;g__;s__ |
| 347195 | forest | 0.8234 | 1 | 0.907 | 0.001 | k__Bacteria;p__Bacteroidetes;c__Sphingobacteriia;o__Sphingobacteriales;f__Sphingobacteriaceae;g__;s__ |
| 878161 | forest | 0.8666 | 0.9444 | 0.905 | 0.001 | k__Bacteria;p__Acidobacteria;c__Acidobacteriia;o__Acidobacteriales;f__Acidobacteriaceae;g__;s__ |
| New.ReferenceOTU83 | forest | 0.8571 | 0.9444 | 0.9 | 0.001 | k__Bacteria;p__Proteobacteria;c__Deltaproteobacteria;o__Myxococcales;f__Cystobacterineae;g__;s__ |
| 226216 | forest | 0.9067 | 0.8889 | 0.898 | 0.001 | k__Bacteria;p__Proteobacteria;c__Betaproteobacteria;o__Burkholderiales;f__Comamonadaceae;g__;s__ |
| New.ReferenceOTU4566 | forest | 0.9041 | 0.8889 | 0.896 | 0.001 | Unassigned;NA;NA;NA;NA;NA;NA |
| 973092 | forest | 0.789 | 1 | 0.888 | 0.001 | k__Bacteria;p__Proteobacteria;c__Deltaproteobacteria;o__Myxococcales;f__Cystobacterineae;g__;s__ |
| 855968 | forest | 0.7834 | 1 | 0.885 | 0.001 | k__Bacteria;p__Proteobacteria;c__Deltaproteobacteria;o__Myxococcales;f__Cystobacterineae;g__;s__ |
| New.ReferenceOTU23 | forest | 0.8775 | 0.8889 | 0.883 | 0.001 | k__Bacteria;p__Proteobacteria;c__Alphaproteobacteria;o__Rhizobiales;f__Methylocystaceae;g__;s__ |
| New.ReferenceOTU4405 | forest | 0.9274 | 0.8333 | 0.879 | 0.001 | Unassigned;NA;NA;NA;NA;NA;NA |
| 979344 | forest | 0.8131 | 0.9444 | 0.876 | 0.001 | k__Bacteria;p__Proteobacteria;c__Alphaproteobacteria;o__Rhizobiales;f__Methylobacteriaceae;g__Methylobacterium;s__ |
| New.ReferenceOTU3 | forest | 0.8093 | 0.9444 | 0.874 | 0.001 | k__Bacteria;p__Proteobacteria;c__Deltaproteobacteria;o__Myxococcales;f__Cystobacterineae;g__;s__ |
| New.ReferenceOTU10 | forest | 0.8578 | 0.8889 | 0.873 | 0.001 | k__Bacteria;p__Proteobacteria;c__Deltaproteobacteria;o__Myxococcales;f__Cystobacterineae;g__;s__ |
| New.ReferenceOTU4768 | forest | 0.9057 | 0.8333 | 0.869 | 0.001 | k__Bacteria;p__Actinobacteria;c__Actinobacteria;o__Actinomycetales;f__Microbacteriaceae;g__;s__ |
| 1008887 | forest | 0.8842 | 0.8333 | 0.858 | 0.001 | k__Bacteria;p__Proteobacteria;c__Alphaproteobacteria;o__Rhizobiales;f__Methylocystaceae;g__;s__ |
| New.ReferenceOTU3914 | forest | 0.9383 | 0.7778 | 0.854 | 0.001 | k__Bacteria;p__Actinobacteria;c__Actinobacteria;o__Actinomycetales;f__Microbacteriaceae;g__;s__ |
| 1087989 | forest | 0.8184 | 0.8889 | 0.853 | 0.001 | k__Bacteria;p__Actinobacteria;c__Actinobacteria;o__Actinomycetales;f__Kineosporiaceae;NA;NA |
| New.ReferenceOTU4 | forest | 0.818 | 0.8889 | 0.853 | 0.001 | k__Bacteria;p__Proteobacteria;c__Alphaproteobacteria;o__Rhodospirillales;f__Acetobacteraceae;g__;s__ |
| 4463045 | forest | 0.93 | 0.7778 | 0.85 | 0.001 | k__Bacteria;p__Proteobacteria;c__Alphaproteobacteria;o__Sphingomonadales;f__Sphingomonadaceae;g__Sphingomonas;s__ |
| 1097610 | forest | 0.8679 | 0.8333 | 0.85 | 0.001 | k__Bacteria;p__Proteobacteria;c__Alphaproteobacteria;o__Rhizobiales;f__Beijerinckiaceae;g__Beijerinckia;s__ |
| New.ReferenceOTU4813 | forest | 1 | 0.7222 | 0.85 | 0.001 | k__Bacteria;p__Proteobacteria;c__Betaproteobacteria;o__Burkholderiales;f__Comamonadaceae;g__;s__ |
| New.ReferenceOTU40 | forest | 0.9269 | 0.7778 | 0.849 | 0.001 | k__Bacteria;p__Bacteroidetes;c__Cytophagia;o__Cytophagales;f__Cytophagaceae;g__;s__ |
| 786067 | forest | 0.8468 | 0.8333 | 0.84 | 0.001 | k__Bacteria;p__[Thermi];c__Deinococci;o__Deinococcales;f__Deinococcaceae;g__Deinococcus;s__ |
| New.ReferenceOTU32 | forest | 0.8397 | 0.8333 | 0.836 | 0.001 | k__Bacteria;p__Actinobacteria;c__Actinobacteria;o__Actinomycetales;f__Nocardioidaceae;g__Friedmanniella;s__ |
| 3692328 | forest | 0.9565 | 0.7222 | 0.831 | 0.001 | k__Bacteria;p__Proteobacteria;c__Alphaproteobacteria;o__Rhizobiales;f__Methylocystaceae;g__;s__ |
| New.ReferenceOTU3455 | forest | 0.9498 | 0.7222 | 0.828 | 0.001 | k__Bacteria;p__Acidobacteria;c__Acidobacteriia;o__Acidobacteriales;f__Acidobacteriaceae;g__;s__ |
| New.ReferenceOTU6216 | forest | 0.9433 | 0.7222 | 0.825 | 0.001 | k__Bacteria;p__Proteobacteria;c__Alphaproteobacteria;o__Rhodospirillales;f__Acetobacteraceae;g__;s__ |
| New.ReferenceOTU25 | forest | 0.813 | 0.8333 | 0.823 | 0.002 | k__Bacteria;p__Proteobacteria;c__Alphaproteobacteria;o__Rhizobiales;f__Methylocystaceae;g__;s__ |
| New.ReferenceOTU4801 | forest | 0.87 | 0.7778 | 0.823 | 0.001 | k__Bacteria;p__Proteobacteria;c__Alphaproteobacteria;o__Rhodospirillales;f__Acetobacteraceae;g__;s__ |
| 704267 | forest | 0.932 | 0.7222 | 0.82 | 0.001 | k__Bacteria;p__Proteobacteria;c__Alphaproteobacteria;o__Sphingomonadales;f__Sphingomonadaceae;g__Sphingomonas;s__ |
| 535429 | forest | 0.8582 | 0.7778 | 0.817 | 0.001 | k__Bacteria;p__Proteobacteria;c__Alphaproteobacteria;o__Rhizobiales;f__Methylocystaceae;g__;s__ |
| New.ReferenceOTU34 | forest | 0.8985 | 0.7222 | 0.806 | 0.001 | k__Bacteria;p__Proteobacteria;c__Alphaproteobacteria;o__Sphingomonadales;f__Sphingomonadaceae;g__Sphingomonas;s__wittichii |
| 1051607 | forest | 0.8873 | 0.7222 | 0.801 | 0.001 | k__Bacteria;p__Proteobacteria;c__Alphaproteobacteria;o__Caulobacterales;f__Caulobacteraceae;g__;s__ |
| 3044098 | forest | 0.8186 | 0.7778 | 0.798 | 0.001 | k__Bacteria;p__Actinobacteria;c__Actinobacteria;o__Actinomycetales;f__Microbacteriaceae;g__;s__ |
| 238508 | forest | 0.8781 | 0.7222 | 0.796 | 0.001 | k__Bacteria;p__Proteobacteria;c__Alphaproteobacteria;o__Sphingomonadales;f__Sphingomonadaceae;g__Sphingomonas;NA |
| New.ReferenceOTU15 | forest | 0.9498 | 0.6667 | 0.796 | 0.001 | k__Bacteria;p__Proteobacteria;c__Deltaproteobacteria;o__Myxococcales;f__Cystobacterineae;g__;s__ |
| 1137871 | forest | 0.8108 | 0.7778 | 0.794 | 0.001 | k__Bacteria;p__Proteobacteria;c__Deltaproteobacteria;o__Myxococcales;f__Cystobacterineae;g__;s__ |
| 4468697 | forest | 0.8065 | 0.7778 | 0.792 | 0.002 | k__Bacteria;p__Bacteroidetes;c__Cytophagia;o__Cytophagales;f__Cytophagaceae;g__Hymenobacter;s__ |
| 1076279 | forest | 0.9161 | 0.6667 | 0.782 | 0.001 | k__Bacteria;p__Bacteroidetes;c__Cytophagia;o__Cytophagales;f__Cytophagaceae;g__Spirosoma;s__ |
| 226138 | forest | 0.8407 | 0.7222 | 0.779 | 0.001 | k__Bacteria;p__Proteobacteria;c__Alphaproteobacteria;o__Sphingomonadales;f__Sphingomonadaceae;g__Sphingomonas;s__ |
| 4310487 | forest | 0.833 | 0.7222 | 0.776 | 0.001 | k__Bacteria;p__Proteobacteria;c__Alphaproteobacteria;o__Sphingomonadales;f__Sphingomonadaceae;g__Sphingomonas;s__ |
| New.ReferenceOTU754 | forest | 0.9757 | 0.6111 | 0.772 | 0.001 | k__Bacteria;p__Proteobacteria;c__Deltaproteobacteria;o__Bdellovibrionales;f__Bdellovibrionaceae;g__Bdellovibrio;s__ |
| 1082552 | forest | 0.8777 | 0.6667 | 0.765 | 0.002 | k__Bacteria;p__Proteobacteria;c__Alphaproteobacteria;o__Rhizobiales;f__Methylobacteriaceae;g__Methylobacterium;s__ |
| New.ReferenceOTU4407 | forest | 0.9446 | 0.6111 | 0.76 | 0.002 | k__Bacteria;p__Proteobacteria;c__Alphaproteobacteria;o__Sphingomonadales;f__Sphingomonadaceae;g__Sphingomonas;s__ |
| 34580 | forest | 0.8639 | 0.6667 | 0.759 | 0.001 | k__Bacteria;p__Proteobacteria;c__Gammaproteobacteria;o__Xanthomonadales;f__Xanthomonadaceae;NA;NA |
| New.ReferenceOTU6142 | forest | 0.8585 | 0.6667 | 0.757 | 0.001 | k__Bacteria;p__Actinobacteria;c__Actinobacteria;o__Actinomycetales;f__Nocardioidaceae;g__Friedmanniella;s__ |
| New.ReferenceOTU6502 | forest | 0.9298 | 0.6111 | 0.754 | 0.001 | k__Bacteria;p__Proteobacteria;c__Deltaproteobacteria;o__Myxococcales;f__Cystobacterineae;g__;s__ |
| 417366 | forest | 0.8418 | 0.6667 | 0.749 | 0.002 | k__Bacteria;p__Proteobacteria;c__Alphaproteobacteria;o__Rhizobiales;f__Methylocystaceae;g__;s__ |
| New.ReferenceOTU56 | forest | 0.917 | 0.6111 | 0.749 | 0.001 | k__Bacteria;p__Proteobacteria;c__Alphaproteobacteria;o__Rhodospirillales;f__Acetobacteraceae;g__;s__ |
| 1039872 | forest | 0.991 | 0.5556 | 0.742 | 0.001 | k__Bacteria;p__Bacteroidetes;c__Cytophagia;o__Cytophagales;f__Cytophagaceae;g__Hymenobacter;s__ |
| 1109067 | forest | 0.9002 | 0.6111 | 0.742 | 0.001 | k__Bacteria;p__Proteobacteria;c__Alphaproteobacteria;o__Rhizobiales;f__Methylobacteriaceae;NA;NA |
| 4472157 | forest | 0.8971 | 0.6111 | 0.74 | 0.002 | k__Bacteria;p__Proteobacteria;c__Alphaproteobacteria;o__Rhizobiales;f__Methylocystaceae;g__;s__ |
| 940662 | forest | 0.8876 | 0.6111 | 0.737 | 0.001 | k__Bacteria;p__FBP;c__;o__;f__;g__;s__ |
| New.ReferenceOTU4809 | forest | 0.971 | 0.5556 | 0.734 | 0.001 | k__Bacteria;p__Proteobacteria;c__Deltaproteobacteria;o__Myxococcales;f__Cystobacterineae;g__;s__ |
| New.ReferenceOTU4508 | forest | 0.9701 | 0.5556 | 0.734 | 0.001 | k__Bacteria;p__Proteobacteria;c__Alphaproteobacteria;o__Rickettsiales;f__;g__;s__ |
| 883987 | forest | 0.8077 | 0.6667 | 0.734 | 0.005 | k__Bacteria;p__Proteobacteria;c__Alphaproteobacteria;o__Sphingomonadales;f__Sphingomonadaceae;g__Sphingomonas;s__wittichii |
| New.ReferenceOTU60 | forest | 0.9621 | 0.5556 | 0.731 | 0.001 | k__Bacteria;p__Proteobacteria;c__Alphaproteobacteria;o__Rickettsiales;f__;g__;s__ |
| 4399333 | forest | 0.8706 | 0.6111 | 0.729 | 0.002 | k__Bacteria;p__Proteobacteria;c__Betaproteobacteria;o__Burkholderiales;f__Oxalobacteraceae;g__;s__ |
| 944401 | forest | 0.7213 | 0.7222 | 0.722 | 0.004 | k__Bacteria;p__Bacteroidetes;c__Sphingobacteriia;o__Sphingobacteriales;f__Sphingobacteriaceae;g__Pedobacter;s__cryoconitis |
| 137634 | forest | 0.9216 | 0.5556 | 0.716 | 0.001 | k__Bacteria;p__FBP;c__;o__;f__;g__;s__ |
| 347428 | forest | 0.768 | 0.6667 | 0.716 | 0.007 | k__Bacteria;p__Proteobacteria;c__Alphaproteobacteria;o__Rhizobiales;f__Hyphomicrobiaceae;g__Devosia;s__ |
| 1085175 | forest | 0.9181 | 0.5556 | 0.714 | 0.001 | k__Bacteria;p__Proteobacteria;c__Deltaproteobacteria;o__Bdellovibrionales;f__Bdellovibrionaceae;g__Bdellovibrio;s__ |
| 1080436 | forest | 0.918 | 0.5556 | 0.714 | 0.001 | k__Bacteria;p__Bacteroidetes;c__Cytophagia;o__Cytophagales;f__Cytophagaceae;g__Hymenobacter;s__ |
| 4425807 | forest | 0.9054 | 0.5556 | 0.709 | 0.001 | k__Bacteria;p__Proteobacteria;c__Alphaproteobacteria;o__Sphingomonadales;f__Sphingomonadaceae;g__Sphingomonas;s__wittichii |
| 4328501 | forest | 0.8209 | 0.6111 | 0.708 | 0.003 | k__Bacteria;p__Acidobacteria;c__Acidobacteriia;o__Acidobacteriales;f__Acidobacteriaceae;g__;s__ |
| 154693 | forest | 0.8116 | 0.6111 | 0.704 | 0.003 | k__Bacteria;p__Proteobacteria;c__Alphaproteobacteria;o__Rickettsiales;f__;g__;s__ |
| 1143820 | forest | 0.743 | 0.6667 | 0.704 | 0.002 | k__Bacteria;p__Proteobacteria;c__Betaproteobacteria;o__Burkholderiales;f__Comamonadaceae;NA;NA |
| New.ReferenceOTU4895 | forest | 0.8895 | 0.5556 | 0.703 | 0.002 | k__Bacteria;p__Actinobacteria;c__Actinobacteria;o__Actinomycetales;f__Pseudonocardiaceae;g__Actinomycetospora;s__ |
| 854941 | vine | 0.8477 | 0.8286 | 0.838 | 0.001 | k__Bacteria;p__Actinobacteria;c__Actinobacteria;o__Actinomycetales;f__Micrococcaceae;g__Micrococcus;s__luteus |
| 1114747 | vine | 0.9895 | 0.6857 | 0.824 | 0.001 | k__Bacteria;p__Proteobacteria;c__Gammaproteobacteria;o__Pseudomonadales;f__Moraxellaceae;g__Enhydrobacter;s__ |
| 1120837 | vine | 0.8539 | 0.7714 | 0.812 | 0.002 | k__Bacteria;p__Firmicutes;c__Bacilli;o__Bacillales;f__Paenibacillaceae;g__Paenibacillus;s__ |
| 4462971 | vine | 0.799 | 0.7429 | 0.77 | 0.003 | k__Bacteria;p__Firmicutes;c__Bacilli;o__Bacillales;f__Paenibacillaceae;g__Paenibacillus;s__ |
| 4425390 | vine | 0.8189 | 0.7143 | 0.765 | 0.001 | k__Bacteria;p__Firmicutes;c__Bacilli;o__Bacillales;f__Bacillaceae;g__Bacillus;s__ |
| 2336242 | vine | 0.7932 | 0.7143 | 0.753 | 0.001 | k__Bacteria;p__Firmicutes;c__Bacilli;o__Bacillales;f__Bacillaceae;g__;s__ |
| 4469492 | vine | 0.8211 | 0.6857 | 0.75 | 0.003 | k__Bacteria;p__Proteobacteria;c__Betaproteobacteria;o__Burkholderiales;f__Comamonadaceae;g__Delftia;s__ |
| 4323871 | vine | 0.7867 | 0.7143 | 0.75 | 0.004 | k__Bacteria;p__Proteobacteria;c__Alphaproteobacteria;o__Rhizobiales;f__Methylobacteriaceae;g__Methylobacterium;s__komagatae |
| 4395697 | vine | 0.9037 | 0.6 | 0.736 | 0.004 | k__Bacteria;p__Proteobacteria;c__Alphaproteobacteria;o__Rhodobacterales;f__Rhodobacteraceae;g__Paracoccus;s__aminovorans |
| 225425 | vine | 0.9036 | 0.6 | 0.736 | 0.001 | k__Bacteria;p__Actinobacteria;c__Acidimicrobiia;o__Acidimicrobiales;f__;g__;s__ |
| 4470837 | vine | 0.8707 | 0.6 | 0.723 | 0.005 | k__Bacteria;p__Firmicutes;c__Bacilli;o__Bacillales;f__Paenibacillaceae;g__Paenibacillus;s__ |
| 4444046 | vine | 0.8271 | 0.6286 | 0.721 | 0.006 | k__Bacteria;p__Actinobacteria;c__Acidimicrobiia;o__Acidimicrobiales;f__;g__;s__ |
| 2616888 | vine | 0.8242 | 0.6286 | 0.72 | 0.007 | k__Bacteria;p__Actinobacteria;c__Actinobacteria;o__Actinomycetales;f__Micromonosporaceae;NA;NA |
| 4404401 | vine | 0.8458 | 0.6 | 0.712 | 0.005 | k__Bacteria;p__Firmicutes;c__Bacilli;o__Bacillales;f__Staphylococcaceae;g__Jeotgalicoccus;s__psychrophilus |
