# Supplementary material for: Shared and unique features of bacterial communities in native forest and vineyard phyllosphere
Source: Ecol Evol. 2019 Feb 20;9(6):3295–305. doi: 10.1002/ece3.4949 (PMC6434556; doi:10.1002/ece3.4949)
Supplement: Supplementary file 7 [file ECE3-9-3295-s007.docx]

Supplementary Table S4. Indicator OTUs of grape leaf vs. grape berry

| OTU ID | group | A | B | stat | p.value | Taxonomy |
| --- | --- | --- | --- | --- | --- | --- |
| 827612 | grape leaf | 0.8959 | 0.8824 | 0.889 | 0.001 | k__Bacteria;p__Proteobacteria;c__Betaproteobacteria;o__Burkholderiales;f__Comamonadaceae;NA;NA |
| 816291 | grape leaf | 1 | 0.7647 | 0.874 | 0.001 | k__Bacteria;p__Actinobacteria;c__Thermoleophilia;o__Gaiellales;f__Gaiellaceae;g__;s__ |
| 4389828 | grape leaf | 0.9047 | 0.8235 | 0.863 | 0.001 | k__Bacteria;p__Actinobacteria;c__Actinobacteria;o__Actinomycetales;f__Streptomycetaceae;g__Streptomyces;s__ |
| 829373 | grape leaf | 0.913 | 0.7647 | 0.836 | 0.001 | k__Bacteria;p__Actinobacteria;c__Actinobacteria;o__Actinomycetales;f__Pseudonocardiaceae;g__Actinomycetospora;s__ |
| 983990 | grape leaf | 0.901 | 0.7647 | 0.83 | 0.001 | k__Bacteria;p__Bacteroidetes;c__Sphingobacteriia;o__Sphingobacteriales;f__Sphingobacteriaceae;g__Pedobacter;s__ |
| 1656470 | grape leaf | 0.8816 | 0.7647 | 0.821 | 0.001 | k__Bacteria;p__Proteobacteria;c__Alphaproteobacteria;o__Rhizobiales;f__Hyphomicrobiaceae;g__Devosia;s__ |
| 4457195 | grape leaf | 0.7513 | 0.8824 | 0.814 | 0.003 | k__Bacteria;p__Bacteroidetes;c__Sphingobacteriia;o__Sphingobacteriales;f__Sphingobacteriaceae;g__;s__ |
| New.ReferenceOTU163 | grape leaf | 0.8545 | 0.7647 | 0.808 | 0.001 | k__Bacteria;p__Proteobacteria;c__Alphaproteobacteria;o__Rhizobiales;f__Hyphomicrobiaceae;g__Devosia;s__ |
| 253735 | grape leaf | 0.7931 | 0.8235 | 0.808 | 0.001 | k__Bacteria;p__Proteobacteria;c__Alphaproteobacteria;o__Rhodospirillales;f__Acetobacteraceae;g__;s__ |
| 4477719 | grape leaf | 0.7597 | 0.8235 | 0.791 | 0.002 | k__Bacteria;p__Proteobacteria;c__Gammaproteobacteria;o__Enterobacteriales;f__Enterobacteriaceae;g__;s__ |
| 4352798 | grape leaf | 0.811 | 0.7647 | 0.788 | 0.004 | k__Bacteria;p__Actinobacteria;c__Actinobacteria;o__Actinomycetales;f__Nocardioidaceae;g__;s__ |
| 1703428 | grape leaf | 0.8049 | 0.7647 | 0.785 | 0.002 | k__Bacteria;p__Actinobacteria;c__Actinobacteria;o__Actinomycetales;f__Micromonosporaceae;g__Pilimelia;s__ |
| 4436954 | grape leaf | 0.785 | 0.7647 | 0.775 | 0.002 | k__Bacteria;p__Proteobacteria;c__Alphaproteobacteria;o__Rhizobiales;f__Hyphomicrobiaceae;g__Devosia;s__ |
| New.ReferenceOTU25 | grape leaf | 0.8462 | 0.7059 | 0.773 | 0.001 | k__Bacteria;p__Proteobacteria;c__Alphaproteobacteria;o__Rhizobiales;f__Methylocystaceae;g__;s__ |
| 235898 | grape leaf | 0.7806 | 0.7647 | 0.773 | 0.004 | k__Bacteria;p__Actinobacteria;c__Actinobacteria;o__Actinomycetales;f__Corynebacteriaceae;g__Corynebacterium;s__ |
| 1111506 | grape leaf | 0.7727 | 0.7647 | 0.769 | 0.004 | k__Bacteria;p__Firmicutes;c__Bacilli;o__Lactobacillales;f__Aerococcaceae;g__;s__ |
| 114201 | grape leaf | 0.7148 | 0.8235 | 0.767 | 0.004 | k__Bacteria;p__Proteobacteria;c__Alphaproteobacteria;o__Rhodospirillales;f__Rhodospirillaceae;g__;s__ |
| 4453998 | grape leaf | 0.7695 | 0.7647 | 0.767 | 0.006 | k__Bacteria;p__Proteobacteria;c__Betaproteobacteria;o__Burkholderiales;f__Comamonadaceae;g__Comamonas;s__ |
| 1127215 | grape leaf | 0.7628 | 0.7647 | 0.764 | 0.003 | k__Bacteria;p__Actinobacteria;c__Actinobacteria;o__Actinomycetales;f__Pseudonocardiaceae;g__Saccharopolyspora;s__ |
| 4328501 | grape leaf | 0.8871 | 0.6471 | 0.758 | 0.002 | k__Bacteria;p__Acidobacteria;c__Acidobacteriia;o__Acidobacteriales;f__Acidobacteriaceae;g__;s__ |
| New.ReferenceOTU6529 | grape leaf | 0.8692 | 0.6471 | 0.75 | 0.004 | Unassigned;NA;NA;NA;NA;NA;NA |
| 4449659 | grape leaf | 0.7952 | 0.7059 | 0.749 | 0.002 | k__Bacteria;p__Actinobacteria;c__Actinobacteria;o__Actinomycetales;f__Nocardiaceae;g__Nocardia;s__ |
| 129622 | grape leaf | 0.6813 | 0.8235 | 0.749 | 0.009 | k__Bacteria;p__Actinobacteria;c__Actinobacteria;o__Actinomycetales;NA;NA;NA |
| 234044 | grape leaf | 0.731 | 0.7647 | 0.748 | 0.004 | k__Bacteria;p__Proteobacteria;c__Betaproteobacteria;o__Burkholderiales;f__Alcaligenaceae;g__Achromobacter;s__ |
| New.ReferenceOTU102 | grape leaf | 0.7246 | 0.7647 | 0.744 | 0.01 | k__Bacteria;p__Firmicutes;c__Bacilli;o__Bacillales;f__Bacillaceae;g__Bacillus;s__ |
| New.ReferenceOTU73 | grape leaf | 0.7246 | 0.7647 | 0.744 | 0.004 | k__Bacteria;p__Proteobacteria;c__Alphaproteobacteria;o__Rhodospirillales;f__Rhodospirillaceae;g__;s__ |
| 156477 | grape leaf | 0.7688 | 0.7059 | 0.737 | 0.012 | k__Bacteria;p__Actinobacteria;c__Actinobacteria;o__Actinomycetales;f__Geodermatophilaceae;g__;s__ |
| 4472437 | grape leaf | 0.8382 | 0.6471 | 0.736 | 0.004 | k__Bacteria;p__Gemmatimonadetes;c__Gemm-3;o__;f__;g__;s__ |
| 1127702 | grape leaf | 0.7652 | 0.7059 | 0.735 | 0.002 | k__Bacteria;p__Actinobacteria;c__Actinobacteria;o__Actinomycetales;f__Microbacteriaceae;g__Leucobacter;s__ |
| 904568 | grape leaf | 0.7469 | 0.7059 | 0.726 | 0.01 | k__Bacteria;p__Proteobacteria;c__Alphaproteobacteria;o__Rhodospirillales;f__Acetobacteraceae;g__;s__ |
| 4437581 | grape leaf | 0.7355 | 0.7059 | 0.721 | 0.007 | k__Bacteria;p__Proteobacteria;c__Alphaproteobacteria;o__Sphingomonadales;f__Sphingomonadaceae;g__Kaistobacter;s__ |
| 968555 | grape leaf | 0.8746 | 0.5882 | 0.717 | 0.003 | k__Bacteria;p__Actinobacteria;c__Actinobacteria;o__Actinomycetales;f__Pseudonocardiaceae;g__Pseudonocardia;s__ |
| 821690 | grape leaf | 0.7934 | 0.6471 | 0.717 | 0.021 | k__Bacteria;p__Proteobacteria;c__Betaproteobacteria;o__Burkholderiales;f__Burkholderiaceae;g__;s__ |
| 864304 | grape leaf | 0.727 | 0.7059 | 0.716 | 0.014 | k__Bacteria;p__Actinobacteria;c__Thermoleophilia;o__Solirubrobacterales;f__;g__;s__ |
| 4390410 | grape leaf | 0.7918 | 0.6471 | 0.716 | 0.004 | k__Bacteria;p__Proteobacteria;c__Gammaproteobacteria;o__Enterobacteriales;f__Enterobacteriaceae;g__;s__ |
| New.ReferenceOTU135 | grape leaf | 0.9657 | 0.5294 | 0.715 | 0.001 | k__Bacteria;p__Proteobacteria;c__Alphaproteobacteria;o__Rhodospirillales;f__Acetobacteraceae;g__;s__ |
| 1069437 | grape leaf | 0.7234 | 0.7059 | 0.715 | 0.01 | k__Bacteria;p__Actinobacteria;c__Thermoleophilia;o__Solirubrobacterales;f__;g__;s__ |
| 55750 | grape leaf | 0.723 | 0.7059 | 0.714 | 0.014 | k__Bacteria;p__Actinobacteria;c__Actinobacteria;o__Actinomycetales;f__Intrasporangiaceae;g__;s__ |
