# Supplementary material for: Shared and unique features of bacterial communities in native forest and vineyard phyllosphere
Source: Ecol Evol. 2019 Feb 20;9(6):3295–305. doi: 10.1002/ece3.4949 (PMC6434556; doi:10.1002/ece3.4949)
Supplement: Supplementary file 8 [file ECE3-9-3295-s008.docx]

Supplementary Table S5. Indicator OTUs of conventional grape lead vs. organic grape leaf

| OTU ID | group | A | B | stat | p.value | Taonomy |
| --- | --- | --- | --- | --- | --- | --- |
| 4409398 | conventional | 0.9351 | 1 | 0.967 | 0.001 | k__Bacteria;p__Proteobacteria;c__Betaproteobacteria;o__Burkholderiales;f__Comamonadaceae;g__Methylibium;s__ |
| 893232 | conventional | 0.8369 | 1 | 0.915 | 0.001 | k__Bacteria;p__Proteobacteria;c__Alphaproteobacteria;o__Rhodospirillales;f__;g__;s__ |
| 4472356 | conventional | 0.9365 | 0.8889 | 0.912 | 0.002 | k__Bacteria;p__Actinobacteria;c__Acidimicrobiia;o__Acidimicrobiales;f__;g__;s__ |
| 531300 | conventional | 0.9256 | 0.8889 | 0.907 | 0.001 | k__Bacteria;p__Actinobacteria;c__Actinobacteria;o__Actinomycetales;f__Brevibacteriaceae;g__Brevibacterium;s__ |
| 4483958 | conventional | 0.8166 | 1 | 0.904 | 0.001 | k__Bacteria;p__Actinobacteria;c__Thermoleophilia;o__Solirubrobacterales;f__;g__;s__ |
| 4305234 | conventional | 0.9185 | 0.8889 | 0.904 | 0.002 | k__Bacteria;p__Actinobacteria;c__Actinobacteria;o__Actinomycetales;f__Propionibacteriaceae;g__;s__ |
| New.ReferenceOTU439 | conventional | 0.9144 | 0.8889 | 0.902 | 0.003 | k__Bacteria;p__Actinobacteria;c__Actinobacteria;o__Actinomycetales;NA;NA;NA |
| 1145616 | conventional | 0.805 | 1 | 0.897 | 0.002 | k__Bacteria;p__Actinobacteria;c__Actinobacteria;o__Actinomycetales;f__Geodermatophilaceae;g__;s__ |
| New.ReferenceOTU170 | conventional | 0.9032 | 0.8889 | 0.896 | 0.003 | k__Bacteria;p__Actinobacteria;c__Actinobacteria;o__Actinomycetales;f__Frankiaceae;g__;s__ |
| 979344 | conventional | 0.7915 | 1 | 0.89 | 0.002 | k__Bacteria;p__Proteobacteria;c__Alphaproteobacteria;o__Rhizobiales;f__Methylobacteriaceae;g__Methylobacterium;s__ |
| 4350881 | conventional | 0.7853 | 1 | 0.886 | 0.007 | k__Bacteria;p__Actinobacteria;c__Actinobacteria;o__Actinomycetales;f__Cellulomonadaceae;NA;NA |
| New.ReferenceOTU4635 | conventional | 0.8815 | 0.8889 | 0.885 | 0.003 | k__Bacteria;p__Actinobacteria;c__Actinobacteria;o__Actinomycetales;f__Geodermatophilaceae;g__;s__ |
| 149506 | conventional | 1 | 0.7778 | 0.882 | 0.004 | k__Bacteria;p__TM7;c__TM7-3;o__;f__;g__;s__ |
| 218567 | conventional | 1 | 0.7778 | 0.882 | 0.003 | k__Bacteria;p__Proteobacteria;c__Deltaproteobacteria;o__Myxococcales;f__;g__;s__ |
| 1127112 | conventional | 1 | 0.7778 | 0.882 | 0.006 | k__Bacteria;p__Bacteroidetes;c__Cytophagia;o__Cytophagales;f__Cytophagaceae;g__Adhaeribacter;s__ |
| 4415788 | conventional | 1 | 0.7778 | 0.882 | 0.001 | k__Bacteria;p__Actinobacteria;c__Actinobacteria;o__Actinomycetales;f__Microbacteriaceae;NA;NA |
| New.ReferenceOTU5726 | conventional | 0.8717 | 0.8889 | 0.88 | 0.003 | k__Bacteria;p__Actinobacteria;c__Actinobacteria;o__Actinomycetales;f__Geodermatophilaceae;NA;NA |
| 409932 | conventional | 0.87 | 0.8889 | 0.879 | 0.003 | k__Bacteria;p__Actinobacteria;c__Actinobacteria;o__Actinomycetales;f__Micromonosporaceae;g__;s__ |
| 241487 | conventional | 0.9504 | 0.7778 | 0.86 | 0.004 | k__Bacteria;p__Actinobacteria;c__Actinobacteria;o__Actinomycetales;f__Streptomycetaceae;g__;s__ |
| New.ReferenceOTU75 | conventional | 0.8305 | 0.8889 | 0.859 | 0.014 | k__Bacteria;p__Proteobacteria;c__Alphaproteobacteria;o__Rhizobiales;f__Bradyrhizobiaceae;g__Balneimonas;s__ |
| New.ReferenceOTU3071 | conventional | 0.9482 | 0.7778 | 0.859 | 0.01 | k__Bacteria;p__Firmicutes;c__Bacilli;o__Bacillales;f__Bacillaceae;g__Bacillus;s__ |
| New.ReferenceOTU25 | conventional | 0.7374 | 1 | 0.859 | 0.012 | k__Bacteria;p__Proteobacteria;c__Alphaproteobacteria;o__Rhizobiales;f__Methylocystaceae;g__;s__ |
| 4083693 | conventional | 0.8255 | 0.8889 | 0.857 | 0.008 | k__Bacteria;p__Actinobacteria;c__Acidimicrobiia;o__Acidimicrobiales;f__C111;g__;s__ |
| New.ReferenceOTU4890 | conventional | 0.9359 | 0.7778 | 0.853 | 0.01 | k__Bacteria;p__Actinobacteria;c__Actinobacteria;o__Actinomycetales;f__Micrococcaceae;NA;NA |
| 4369416 | conventional | 0.926 | 0.7778 | 0.849 | 0.006 | k__Bacteria;p__Actinobacteria;c__Actinobacteria;o__Actinomycetales;f__Micrococcaceae;g__;s__ |
| 4472969 | conventional | 0.7175 | 1 | 0.847 | 0.017 | k__Bacteria;p__Proteobacteria;c__Deltaproteobacteria;o__Myxococcales;f__;g__;s__ |
| New.ReferenceOTU4640 | conventional | 0.9202 | 0.7778 | 0.846 | 0.012 | k__Bacteria;p__Proteobacteria;c__Alphaproteobacteria;o__Rhodospirillales;f__Rhodospirillaceae;g__;s__ |
| New.ReferenceOTU1489 | conventional | 0.9194 | 0.7778 | 0.846 | 0.008 | k__Bacteria;p__Actinobacteria;c__Actinobacteria;o__Actinomycetales;f__Geodermatophilaceae;NA;NA |
| New.ReferenceOTU486 | conventional | 0.7957 | 0.8889 | 0.841 | 0.015 | k__Bacteria;p__Firmicutes;c__Clostridia;o__Clostridiales;NA;NA;NA |
| New.ReferenceOTU2620 | conventional | 0.9029 | 0.7778 | 0.838 | 0.008 | k__Bacteria;p__Firmicutes;c__Clostridia;o__Clostridiales;f__Peptostreptococcaceae;NA;NA |
| New.ReferenceOTU355 | conventional | 0.8997 | 0.7778 | 0.836 | 0.008 | k__Bacteria;p__Proteobacteria;c__Alphaproteobacteria;o__Rhizobiales;f__Methylobacteriaceae;g__Methylobacterium;s__adhaesivum |
| New.ReferenceOTU4307 | conventional | 0.7851 | 0.8889 | 0.835 | 0.015 | k__Bacteria;p__Firmicutes;c__Bacilli;o__Bacillales;f__Bacillaceae;g__Bacillus;s__ |
| New.ReferenceOTU4878 | conventional | 0.8971 | 0.7778 | 0.835 | 0.013 | k__Bacteria;p__Firmicutes;c__Bacilli;o__Bacillales;f__Planococcaceae;g__Sporosarcina;s__ |
| New.ReferenceOTU6512 | conventional | 0.897 | 0.7778 | 0.835 | 0.013 | k__Bacteria;p__Gemmatimonadetes;c__Gemmatimonadetes;o__Gemmatimonadales;f__;g__;s__ |
| New.ReferenceOTU4942 | conventional | 0.8924 | 0.7778 | 0.833 | 0.008 | k__Bacteria;p__Actinobacteria;c__Actinobacteria;o__Actinomycetales;f__Geodermatophilaceae;g__;s__ |
| 4359936 | conventional | 0.8896 | 0.7778 | 0.832 | 0.01 | k__Bacteria;p__Firmicutes;c__Bacilli;o__Bacillales;f__Planococcaceae;NA;NA |
| 810425 | conventional | 0.8805 | 0.7778 | 0.828 | 0.009 | k__Bacteria;p__Actinobacteria;c__Actinobacteria;o__Actinomycetales;f__Corynebacteriaceae;g__Corynebacterium;s__ |
| 4442627 | conventional | 0.7674 | 0.8889 | 0.826 | 0.016 | k__Bacteria;p__Proteobacteria;c__Gammaproteobacteria;o__Thiotrichales;f__Piscirickettsiaceae;g__;s__ |
| 4327040 | conventional | 0.7669 | 0.8889 | 0.826 | 0.021 | k__Bacteria;p__Gemmatimonadetes;c__Gemm-5;o__;f__;g__;s__ |
| 1097610 | conventional | 0.7657 | 0.8889 | 0.825 | 0.015 | k__Bacteria;p__Proteobacteria;c__Alphaproteobacteria;o__Rhizobiales;f__Beijerinckiaceae;g__Beijerinckia;s__ |
| New.ReferenceOTU1224 | conventional | 0.7643 | 0.8889 | 0.824 | 0.031 | k__Bacteria;p__Firmicutes;c__Bacilli;o__Bacillales;f__Bacillaceae;g__Bacillus;s__ |
| 4328501 | conventional | 0.7636 | 0.8889 | 0.824 | 0.023 | k__Bacteria;p__Acidobacteria;c__Acidobacteriia;o__Acidobacteriales;f__Acidobacteriaceae;g__;s__ |
| 863942 | conventional | 0.7613 | 0.8889 | 0.823 | 0.024 | k__Bacteria;p__Proteobacteria;c__Alphaproteobacteria;o__Rhizobiales;f__Methylocystaceae;g__;s__ |
| 543740 | conventional | 0.7608 | 0.8889 | 0.822 | 0.031 | k__Bacteria;p__Actinobacteria;c__Actinobacteria;o__Actinomycetales;f__Sporichthyaceae;g__;s__ |
| 4464969 | conventional | 0.868 | 0.7778 | 0.822 | 0.016 | k__Bacteria;p__Firmicutes;c__Clostridia;o__Clostridiales;f__Ruminococcaceae;g__;s__ |
| 225425 | conventional | 0.8646 | 0.7778 | 0.82 | 0.017 | k__Bacteria;p__Actinobacteria;c__Acidimicrobiia;o__Acidimicrobiales;f__;g__;s__ |
| 4476648 | conventional | 1 | 0.6667 | 0.816 | 0.014 | k__Bacteria;p__Proteobacteria;c__Betaproteobacteria;o__Burkholderiales;f__Comamonadaceae;g__;s__ |
| 4391050 | conventional | 1 | 0.6667 | 0.816 | 0.008 | k__Bacteria;p__Actinobacteria;c__Actinobacteria;o__Actinomycetales;f__Propionibacteriaceae;g__;s__ |
| 161199 | conventional | 1 | 0.6667 | 0.816 | 0.005 | k__Bacteria;p__Actinobacteria;c__Actinobacteria;o__Actinomycetales;f__Micromonosporaceae;g__;s__ |
| 4303162 | conventional | 1 | 0.6667 | 0.816 | 0.012 | k__Bacteria;p__Actinobacteria;c__Actinobacteria;o__Actinomycetales;f__Pseudonocardiaceae;g__Pseudonocardia;s__ |
| 153548 | conventional | 1 | 0.6667 | 0.816 | 0.005 | k__Bacteria;p__Actinobacteria;c__Actinobacteria;o__Actinomycetales;f__;g__;s__ |
| 4435575 | conventional | 1 | 0.6667 | 0.816 | 0.011 | k__Bacteria;p__Firmicutes;c__Clostridia;o__Clostridiales;f__;g__;s__ |
| 18516 | conventional | 1 | 0.6667 | 0.816 | 0.012 | k__Bacteria;p__Proteobacteria;c__Gammaproteobacteria;o__Xanthomonadales;f__Sinobacteraceae;g__;s__ |
| 304089 | conventional | 1 | 0.6667 | 0.816 | 0.005 | k__Bacteria;p__Actinobacteria;c__Thermoleophilia;o__Solirubrobacterales;f__Patulibacteraceae;g__;s__ |
| 1142524 | conventional | 1 | 0.6667 | 0.816 | 0.008 | k__Bacteria;p__Firmicutes;c__Bacilli;o__Bacillales;f__Bacillaceae;g__;s__ |
| 244375 | conventional | 1 | 0.6667 | 0.816 | 0.013 | k__Bacteria;p__Actinobacteria;c__Thermoleophilia;o__Solirubrobacterales;f__;g__;s__ |
| 1005794 | conventional | 1 | 0.6667 | 0.816 | 0.015 | k__Bacteria;p__Actinobacteria;c__Acidimicrobiia;o__Acidimicrobiales;f__;g__;s__ |
| New.ReferenceOTU3026 | conventional | 1 | 0.6667 | 0.816 | 0.015 | k__Bacteria;p__Firmicutes;c__Bacilli;o__Bacillales;f__Bacillaceae;g__Bacillus;s__ |
| New.ReferenceOTU593 | conventional | 1 | 0.6667 | 0.816 | 0.012 | k__Bacteria;p__Actinobacteria;c__Actinobacteria;o__Actinomycetales;f__Micrococcaceae;g__;s__ |
| New.ReferenceOTU5141 | conventional | 1 | 0.6667 | 0.816 | 0.012 | k__Bacteria;p__Actinobacteria;c__Actinobacteria;o__Actinomycetales;f__Micrococcaceae;g__Microbispora;s__rosea |
| New.ReferenceOTU2117 | conventional | 1 | 0.6667 | 0.816 | 0.007 | k__Bacteria;p__Firmicutes;c__Bacilli;o__Bacillales;f__Bacillaceae;g__Bacillus;s__ |
| New.ReferenceOTU1597 | conventional | 1 | 0.6667 | 0.816 | 0.006 | k__Bacteria;p__Actinobacteria;c__Actinobacteria;o__Actinomycetales;f__Micromonosporaceae;g__;s__ |
| New.ReferenceOTU2416 | conventional | 1 | 0.6667 | 0.816 | 0.007 | k__Bacteria;p__Actinobacteria;c__Actinobacteria;o__Actinomycetales;f__Cellulomonadaceae;NA;NA |
| New.ReferenceOTU543 | conventional | 1 | 0.6667 | 0.816 | 0.012 | k__Bacteria;p__Actinobacteria;c__Actinobacteria;o__Actinomycetales;NA;NA;NA |
| New.ReferenceOTU198 | conventional | 1 | 0.6667 | 0.816 | 0.007 | k__Bacteria;p__Actinobacteria;c__Thermoleophilia;o__Gaiellales;f__;g__;s__ |
| New.ReferenceOTU301 | conventional | 1 | 0.6667 | 0.816 | 0.012 | k__Bacteria;p__Firmicutes;c__Bacilli;o__Bacillales;f__Planococcaceae;g__Solibacillus;s__ |
| New.ReferenceOTU143 | conventional | 1 | 0.6667 | 0.816 | 0.014 | k__Bacteria;p__Bacteroidetes;c__Cytophagia;o__Cytophagales;f__Cytophagaceae;g__Pontibacter;s__ |
| New.ReferenceOTU2509 | conventional | 1 | 0.6667 | 0.816 | 0.012 | k__Bacteria;p__Actinobacteria;c__Thermoleophilia;o__Gaiellales;f__Gaiellaceae;g__;s__ |
| New.ReferenceOTU3318 | conventional | 1 | 0.6667 | 0.816 | 0.015 | k__Bacteria;p__Firmicutes;c__Bacilli;o__Bacillales;f__Planococcaceae;g__Sporosarcina;s__ginsengi |
| New.ReferenceOTU1434 | conventional | 1 | 0.6667 | 0.816 | 0.012 | k__Bacteria;p__Firmicutes;c__Bacilli;o__Bacillales;f__Bacillaceae;g__Bacillus;s__ |
| New.ReferenceOTU1725 | conventional | 1 | 0.6667 | 0.816 | 0.008 | k__Bacteria;p__Actinobacteria;c__Actinobacteria;o__Actinomycetales;NA;NA;NA |
| 229359 | conventional | 0.7497 | 0.8889 | 0.816 | 0.021 | k__Bacteria;p__Proteobacteria;c__Alphaproteobacteria;o__Rhodospirillales;f__Acetobacteraceae;g__Gluconobacter;s__ |
| New.ReferenceOTU4325 | conventional | 0.8484 | 0.7778 | 0.812 | 0.018 | k__Bacteria;p__Actinobacteria;c__Actinobacteria;o__Actinomycetales;f__Cellulomonadaceae;g__Cellulomonas;s__ |
| 1062704 | conventional | 0.7413 | 0.8889 | 0.812 | 0.03 | k__Bacteria;p__Firmicutes;c__Bacilli;o__Bacillales;NA;NA;NA |
| New.ReferenceOTU567 | conventional | 0.8428 | 0.7778 | 0.81 | 0.037 | k__Bacteria;p__Firmicutes;c__Bacilli;o__Bacillales;f__Bacillaceae;g__Bacillus;s__ |
| New.ReferenceOTU2089 | conventional | 0.8422 | 0.7778 | 0.809 | 0.017 | k__Bacteria;p__Actinobacteria;c__Actinobacteria;o__Actinomycetales;f__Micromonosporaceae;g__;s__ |
| 689528 | conventional | 0.8382 | 0.7778 | 0.807 | 0.019 | k__Bacteria;p__Actinobacteria;c__Actinobacteria;o__Actinomycetales;f__Intrasporangiaceae;g__;s__ |
| New.ReferenceOTU367 | conventional | 0.8376 | 0.7778 | 0.807 | 0.02 | k__Bacteria;p__Actinobacteria;c__Actinobacteria;o__Actinomycetales;f__Micromonosporaceae;NA;NA |
| 4393701 | conventional | 0.8372 | 0.7778 | 0.807 | 0.019 | k__Bacteria;p__Proteobacteria;c__Betaproteobacteria;o__Burkholderiales;f__Oxalobacteraceae;NA;NA |
| 4395995 | conventional | 0.7312 | 0.8889 | 0.806 | 0.032 | k__Bacteria;p__Proteobacteria;c__Betaproteobacteria;o__Burkholderiales;f__Comamonadaceae;g__;s__ |
| 1029334 | conventional | 0.8342 | 0.7778 | 0.805 | 0.024 | k__Bacteria;p__Bacteroidetes;c__[Saprospirae];o__[Saprospirales];f__Chitinophagaceae;g__Segetibacter;s__ |
| 580625 | conventional | 0.8265 | 0.7778 | 0.802 | 0.022 | k__Bacteria;p__Proteobacteria;c__Alphaproteobacteria;o__Rhizobiales;f__Bradyrhizobiaceae;g__;s__ |
| New.ReferenceOTU929 | conventional | 0.8198 | 0.7778 | 0.799 | 0.019 | k__Bacteria;p__Firmicutes;c__Bacilli;o__Bacillales;f__Bacillaceae;g__Bacillus;s__ |
| New.ReferenceOTU745 | conventional | 0.9538 | 0.6667 | 0.797 | 0.017 | k__Bacteria;p__Actinobacteria;c__Actinobacteria;o__Actinomycetales;f__Geodermatophilaceae;g__;s__ |
| 4371107 | conventional | 0.8101 | 0.7778 | 0.794 | 0.037 | k__Bacteria;p__Actinobacteria;c__Thermoleophilia;o__Solirubrobacterales;f__Conexibacteraceae;g__Conexibacter;s__ |
| 725990 | conventional | 0.8062 | 0.7778 | 0.792 | 0.024 | k__Bacteria;p__Actinobacteria;c__Actinobacteria;o__Actinomycetales;f__Micrococcaceae;g__Micrococcus;s__luteus |
| 4185921 | conventional | 0.7044 | 0.8889 | 0.791 | 0.035 | k__Bacteria;p__Proteobacteria;c__Alphaproteobacteria;o__Sphingomonadales;f__Sphingomonadaceae;g__Sphingomonas;s__ |
| 4303161 | conventional | 0.8015 | 0.7778 | 0.79 | 0.021 | k__Bacteria;p__Actinobacteria;c__Actinobacteria;o__Actinomycetales;f__Pseudonocardiaceae;g__Pseudonocardia;s__ |
| 1125443 | conventional | 0.933 | 0.6667 | 0.789 | 0.024 | k__Bacteria;p__Firmicutes;c__Bacilli;o__Bacillales;f__Planococcaceae;g__;s__ |
| New.ReferenceOTU3846 | conventional | 0.9326 | 0.6667 | 0.789 | 0.033 | k__Bacteria;p__Actinobacteria;c__Actinobacteria;o__Actinomycetales;f__Microbacteriaceae;g__;s__ |
| New.ReferenceOTU5832 | conventional | 0.7966 | 0.7778 | 0.787 | 0.037 | k__Bacteria;p__Actinobacteria;c__Actinobacteria;o__Actinomycetales;f__Micromonosporaceae;g__;s__ |
| New.ReferenceOTU655 | conventional | 0.7936 | 0.7778 | 0.786 | 0.023 | k__Bacteria;p__Actinobacteria;c__Actinobacteria;o__Actinomycetales;f__Geodermatophilaceae;g__;s__ |
| New.ReferenceOTU2193 | conventional | 0.9223 | 0.6667 | 0.784 | 0.027 | k__Bacteria;p__Firmicutes;c__Bacilli;o__Bacillales;f__Bacillaceae;g__Bacillus;s__ |
| New.ReferenceOTU2619 | conventional | 0.9187 | 0.6667 | 0.783 | 0.024 | k__Bacteria;p__Actinobacteria;c__Actinobacteria;o__Actinomycetales;f__Micromonosporaceae;NA;NA |
| New.ReferenceOTU1092 | conventional | 0.7859 | 0.7778 | 0.782 | 0.033 | k__Bacteria;p__Actinobacteria;c__Actinobacteria;o__Actinomycetales;f__Pseudonocardiaceae;g__Pseudonocardia;s__ |
| 4335725 | conventional | 0.784 | 0.7778 | 0.781 | 0.042 | k__Bacteria;p__Proteobacteria;c__Betaproteobacteria;o__Burkholderiales;f__Comamonadaceae;NA;NA |
| 3151583 | conventional | 0.7824 | 0.7778 | 0.78 | 0.036 | k__Bacteria;p__Firmicutes;c__Bacilli;o__Bacillales;f__Bacillaceae;g__Bacillus;s__ |
| 191973 | conventional | 0.9108 | 0.6667 | 0.779 | 0.028 | k__Bacteria;p__Actinobacteria;c__Actinobacteria;o__Actinomycetales;f__Micrococcaceae;g__;s__ |
| 4451545 | conventional | 0.7779 | 0.7778 | 0.778 | 0.042 | k__Bacteria;p__Proteobacteria;c__Alphaproteobacteria;o__Rhizobiales;f__Beijerinckiaceae;g__;s__ |
| 4468697 | conventional | 0.7755 | 0.7778 | 0.777 | 0.037 | k__Bacteria;p__Bacteroidetes;c__Cytophagia;o__Cytophagales;f__Cytophagaceae;g__Hymenobacter;s__ |
| 4468466 | conventional | 0.9015 | 0.6667 | 0.775 | 0.047 | k__Bacteria;p__Firmicutes;c__Clostridia;o__Clostridiales;f__Ruminococcaceae;g__;s__ |
| New.ReferenceOTU3703 | conventional | 0.8986 | 0.6667 | 0.774 | 0.032 | k__Bacteria;p__Chloroflexi;c__Chloroflexi;o__[Roseiflexales];f__[Kouleothrixaceae];g__;s__ |
| 247009 | conventional | 0.8983 | 0.6667 | 0.774 | 0.031 | k__Bacteria;p__Firmicutes;c__Bacilli;o__Bacillales;f__Planococcaceae;g__;s__ |
| 4370912 | conventional | 0.7689 | 0.7778 | 0.773 | 0.048 | k__Bacteria;p__Firmicutes;c__Clostridia;o__Clostridiales;f__;g__;s__ |
| New.ReferenceOTU419 | conventional | 0.7686 | 0.7778 | 0.773 | 0.026 | k__Bacteria;p__Proteobacteria;c__Gammaproteobacteria;o__Enterobacteriales;f__Enterobacteriaceae;g__;s__ |
| New.ReferenceOTU6513 | conventional | 0.8871 | 0.6667 | 0.769 | 0.029 | k__Bacteria;p__Actinobacteria;c__Actinobacteria;o__Actinomycetales;f__;g__;s__ |
| New.ReferenceOTU672 | conventional | 0.8867 | 0.6667 | 0.769 | 0.03 | k__Bacteria;p__Proteobacteria;c__Alphaproteobacteria;o__Sphingomonadales;f__Sphingomonadaceae;g__Sphingomonas;s__yabuuchiae |
| New.ReferenceOTU204 | conventional | 0.8864 | 0.6667 | 0.769 | 0.037 | k__Bacteria;p__Actinobacteria;c__Actinobacteria;o__Actinomycetales;f__Micrococcaceae;g__;s__ |
| 3582363 | conventional | 0.7594 | 0.7778 | 0.769 | 0.041 | k__Bacteria;p__Proteobacteria;c__Deltaproteobacteria;o__Myxococcales;f__OM27;g__;s__ |
| New.ReferenceOTU1773 | conventional | 0.8851 | 0.6667 | 0.768 | 0.026 | k__Bacteria;p__Firmicutes;c__Clostridia;o__Clostridiales;f__Clostridiaceae;g__Clostridium;s__ |
| 870470 | conventional | 0.7525 | 0.7778 | 0.765 | 0.045 | k__Bacteria;p__Proteobacteria;c__Alphaproteobacteria;o__Rhizobiales;f__Methylocystaceae;g__;s__ |
| 4443307 | conventional | 0.874 | 0.6667 | 0.763 | 0.032 | k__Bacteria;p__Firmicutes;c__Clostridia;o__Clostridiales;f__Clostridiaceae;g__Clostridium;NA |
| 4441441 | conventional | 0.748 | 0.7778 | 0.763 | 0.04 | k__Bacteria;p__Firmicutes;c__Clostridia;o__Clostridiales;f__[Tissierellaceae];g__;s__ |
| 213580 | conventional | 0.8718 | 0.6667 | 0.762 | 0.03 | k__Bacteria;p__Actinobacteria;c__Thermoleophilia;o__Gaiellales;f__;g__;s__ |
| 883987 | conventional | 0.8703 | 0.6667 | 0.762 | 0.039 | k__Bacteria;p__Proteobacteria;c__Alphaproteobacteria;o__Sphingomonadales;f__Sphingomonadaceae;g__Sphingomonas;s__wittichii |
| New.ReferenceOTU3496 | conventional | 0.8665 | 0.6667 | 0.76 | 0.029 | k__Bacteria;p__Actinobacteria;c__Actinobacteria;o__Actinomycetales;f__Geodermatophilaceae;NA;NA |
| 914962 | conventional | 0.8665 | 0.6667 | 0.76 | 0.029 | k__Bacteria;p__Proteobacteria;c__Alphaproteobacteria;o__Rhodospirillales;f__;g__;s__ |
| New.ReferenceOTU6542 | conventional | 0.8545 | 0.6667 | 0.755 | 0.049 | k__Bacteria;p__Actinobacteria;c__Actinobacteria;o__Actinomycetales;NA;NA;NA |
| 132913 | conventional | 0.8532 | 0.6667 | 0.754 | 0.032 | k__Bacteria;p__Actinobacteria;c__Actinobacteria;o__Actinomycetales;f__Pseudonocardiaceae;g__Pseudonocardia;s__ |
| 4444938 | conventional | 0.8501 | 0.6667 | 0.753 | 0.043 | k__Bacteria;p__Actinobacteria;c__Acidimicrobiia;o__Acidimicrobiales;f__;g__;s__ |
| New.ReferenceOTU5845 | conventional | 0.8455 | 0.6667 | 0.751 | 0.041 | k__Bacteria;p__Actinobacteria;c__Actinobacteria;o__Actinomycetales;f__Geodermatophilaceae;g__;s__ |
| 781021 | conventional | 0.8452 | 0.6667 | 0.751 | 0.038 | k__Bacteria;p__Firmicutes;c__Clostridia;o__;f__;g__;s__ |
| 922149 | conventional | 1 | 0.5556 | 0.745 | 0.025 | k__Bacteria;p__Actinobacteria;c__Acidimicrobiia;o__Acidimicrobiales;f__;g__;s__ |
| 311130 | conventional | 1 | 0.5556 | 0.745 | 0.03 | k__Bacteria;p__Chloroflexi;c__Chloroflexi;o__AKIW781;f__;g__;s__ |
| 580850 | conventional | 1 | 0.5556 | 0.745 | 0.035 | k__Bacteria;p__Actinobacteria;c__Actinobacteria;o__Actinomycetales;f__Micromonosporaceae;g__;s__ |
| 158034 | conventional | 1 | 0.5556 | 0.745 | 0.03 | k__Bacteria;p__Proteobacteria;c__Alphaproteobacteria;o__Sphingomonadales;f__Sphingomonadaceae;g__Sphingomonas;s__wittichii |
| 4008780 | conventional | 1 | 0.5556 | 0.745 | 0.028 | k__Bacteria;p__Proteobacteria;c__Deltaproteobacteria;o__Myxococcales;f__Haliangiaceae;g__;s__ |
| 169737 | conventional | 1 | 0.5556 | 0.745 | 0.041 | k__Bacteria;p__Proteobacteria;c__Betaproteobacteria;o__SC-I-84;f__;g__;s__ |
| 4320368 | conventional | 1 | 0.5556 | 0.745 | 0.04 | k__Bacteria;p__Proteobacteria;c__Betaproteobacteria;o__Burkholderiales;f__Comamonadaceae;NA;NA |
| 44075 | conventional | 1 | 0.5556 | 0.745 | 0.034 | k__Bacteria;p__Bacteroidetes;c__Sphingobacteriia;o__Sphingobacteriales;f__Sphingobacteriaceae;g__Pedobacter;s__ |
| 217328 | conventional | 1 | 0.5556 | 0.745 | 0.027 | k__Bacteria;p__Proteobacteria;c__Deltaproteobacteria;o__Myxococcales;f__Haliangiaceae;g__;s__ |
| 262130 | conventional | 1 | 0.5556 | 0.745 | 0.029 | k__Bacteria;p__Firmicutes;c__Bacilli;o__Bacillales;f__Bacillaceae;g__Bacillus;s__ |
| 909520 | conventional | 1 | 0.5556 | 0.745 | 0.02 | k__Bacteria;p__Proteobacteria;c__Deltaproteobacteria;o__Myxococcales;f__Myxococcaceae;g__Anaeromyxobacter;s__ |
| 4387422 | conventional | 1 | 0.5556 | 0.745 | 0.037 | k__Bacteria;p__Proteobacteria;c__Alphaproteobacteria;o__Rhizobiales;f__Hyphomicrobiaceae;g__Devosia;s__ |
| 1100123 | conventional | 1 | 0.5556 | 0.745 | 0.029 | k__Bacteria;p__Actinobacteria;c__Thermoleophilia;o__Solirubrobacterales;f__Solirubrobacteraceae;g__;s__ |
| 3900488 | conventional | 1 | 0.5556 | 0.745 | 0.037 | k__Bacteria;p__Firmicutes;c__Clostridia;o__Clostridiales;f__Clostridiaceae;g__;s__ |
| 255034 | conventional | 1 | 0.5556 | 0.745 | 0.039 | k__Bacteria;p__Firmicutes;c__Clostridia;o__Clostridiales;NA;NA;NA |
| 4415177 | conventional | 1 | 0.5556 | 0.745 | 0.027 | k__Bacteria;p__Firmicutes;c__Bacilli;o__Lactobacillales;f__Streptococcaceae;g__Streptococcus;s__ |
| 1118409 | conventional | 1 | 0.5556 | 0.745 | 0.035 | k__Bacteria;p__Proteobacteria;c__Deltaproteobacteria;o__Myxococcales;f__;g__;s__ |
| 513591 | conventional | 1 | 0.5556 | 0.745 | 0.026 | k__Bacteria;p__Acidobacteria;c__Solibacteres;o__Solibacterales;f__Solibacteraceae;g__;s__ |
| 4472772 | conventional | 1 | 0.5556 | 0.745 | 0.039 | k__Bacteria;p__Proteobacteria;c__Alphaproteobacteria;o__Rhizobiales;f__Bradyrhizobiaceae;NA;NA |
| 4421014 | conventional | 1 | 0.5556 | 0.745 | 0.038 | k__Bacteria;p__Bacteroidetes;c__[Saprospirae];o__[Saprospirales];f__Chitinophagaceae;g__;s__ |
| 4309509 | conventional | 1 | 0.5556 | 0.745 | 0.027 | k__Bacteria;p__Firmicutes;c__Clostridia;o__Clostridiales;f__Lachnospiraceae;g__Coprococcus;s__ |
| 509891 | conventional | 1 | 0.5556 | 0.745 | 0.028 | k__Bacteria;p__Proteobacteria;c__Alphaproteobacteria;o__Rhodospirillales;f__Acetobacteraceae;g__;s__ |
| 2344719 | conventional | 1 | 0.5556 | 0.745 | 0.037 | k__Bacteria;p__Proteobacteria;c__Alphaproteobacteria;o__Rhizobiales;f__Hyphomicrobiaceae;g__;s__ |
| 4332126 | conventional | 1 | 0.5556 | 0.745 | 0.033 | k__Bacteria;p__Actinobacteria;c__Acidimicrobiia;o__Acidimicrobiales;f__;g__;s__ |
| 952388 | conventional | 1 | 0.5556 | 0.745 | 0.026 | k__Bacteria;p__Proteobacteria;c__Alphaproteobacteria;o__Rhodobacterales;f__Rhodobacteraceae;g__Rubellimicrobium;s__ |
| 831178 | conventional | 1 | 0.5556 | 0.745 | 0.041 | k__Bacteria;p__Proteobacteria;c__Alphaproteobacteria;o__Rhizobiales;f__;g__;s__ |
| 2316421 | conventional | 1 | 0.5556 | 0.745 | 0.032 | k__Bacteria;p__Chloroflexi;c__C0119;o__;f__;g__;s__ |
| 1140016 | conventional | 1 | 0.5556 | 0.745 | 0.034 | k__Bacteria;p__Actinobacteria;c__Actinobacteria;o__Actinomycetales;f__Micromonosporaceae;g__;s__ |
| 4434807 | conventional | 1 | 0.5556 | 0.745 | 0.03 | k__Bacteria;p__Actinobacteria;c__Actinobacteria;o__Actinomycetales;f__Nocardioidaceae;g__;s__ |
| New.ReferenceOTU109 | conventional | 1 | 0.5556 | 0.745 | 0.039 | k__Bacteria;p__Chloroflexi;c__Chloroflexi;o__[Roseiflexales];f__[Kouleothrixaceae];g__;s__ |
| New.ReferenceOTU590 | conventional | 1 | 0.5556 | 0.745 | 0.037 | k__Bacteria;p__Actinobacteria;c__Actinobacteria;o__Actinomycetales;NA;NA;NA |
| New.ReferenceOTU3015 | conventional | 1 | 0.5556 | 0.745 | 0.037 | k__Bacteria;p__Firmicutes;c__Bacilli;o__Bacillales;f__Bacillaceae;g__Bacillus;s__ |
| New.ReferenceOTU605 | conventional | 1 | 0.5556 | 0.745 | 0.034 | k__Bacteria;p__Proteobacteria;c__Deltaproteobacteria;o__Myxococcales;f__Polyangiaceae;g__;s__ |
| New.ReferenceOTU5858 | conventional | 1 | 0.5556 | 0.745 | 0.037 | k__Bacteria;p__Proteobacteria;c__Alphaproteobacteria;o__Rhizobiales;f__Methylobacteriaceae;g__Methylobacterium;s__ |
| New.ReferenceOTU3224 | conventional | 1 | 0.5556 | 0.745 | 0.035 | k__Bacteria;p__Actinobacteria;c__Actinobacteria;o__Actinomycetales;f__Geodermatophilaceae;NA;NA |
| New.ReferenceOTU3032 | conventional | 1 | 0.5556 | 0.745 | 0.026 | k__Bacteria;p__Actinobacteria;c__Actinobacteria;o__Actinomycetales;f__Corynebacteriaceae;g__Corynebacterium;s__ |
| New.ReferenceOTU2861 | conventional | 1 | 0.5556 | 0.745 | 0.038 | k__Bacteria;p__Gemmatimonadetes;c__Gemmatimonadetes;o__Gemmatimonadales;f__;g__;s__ |
| New.ReferenceOTU1386 | conventional | 1 | 0.5556 | 0.745 | 0.034 | k__Bacteria;p__Actinobacteria;c__Actinobacteria;o__Actinomycetales;f__Geodermatophilaceae;g__;s__ |
| New.ReferenceOTU1636 | conventional | 1 | 0.5556 | 0.745 | 0.037 | k__Bacteria;p__Actinobacteria;c__Actinobacteria;o__Actinomycetales;f__Geodermatophilaceae;NA;NA |
| New.ReferenceOTU505 | conventional | 1 | 0.5556 | 0.745 | 0.037 | k__Bacteria;p__Actinobacteria;c__Actinobacteria;o__Actinomycetales;f__Nocardioidaceae;g__Friedmanniella;s__ |
| New.ReferenceOTU2563 | conventional | 1 | 0.5556 | 0.745 | 0.037 | k__Bacteria;p__Firmicutes;c__Bacilli;o__Bacillales;f__Planococcaceae;g__Sporosarcina;s__ |
| New.ReferenceOTU5374 | conventional | 1 | 0.5556 | 0.745 | 0.037 | k__Bacteria;p__Firmicutes;c__Clostridia;o__Clostridiales;f__;g__;s__ |
| New.ReferenceOTU615 | conventional | 1 | 0.5556 | 0.745 | 0.037 | k__Bacteria;p__Firmicutes;c__Bacilli;o__Bacillales;f__Bacillaceae;g__Bacillus;s__ |
| New.ReferenceOTU1332 | conventional | 1 | 0.5556 | 0.745 | 0.034 | k__Bacteria;p__Actinobacteria;c__Actinobacteria;o__Actinomycetales;f__Microbacteriaceae;g__;s__ |
| New.ReferenceOTU3166 | conventional | 1 | 0.5556 | 0.745 | 0.028 | k__Bacteria;p__Actinobacteria;c__Actinobacteria;o__Actinomycetales;f__Micromonosporaceae;g__Actinoplanes;s__ |
| New.ReferenceOTU3295 | conventional | 1 | 0.5556 | 0.745 | 0.034 | k__Bacteria;p__Actinobacteria;c__Actinobacteria;o__Actinomycetales;f__Pseudonocardiaceae;g__Pseudonocardia;s__ |
| New.ReferenceOTU3818 | conventional | 1 | 0.5556 | 0.745 | 0.029 | k__Bacteria;p__Actinobacteria;c__Actinobacteria;o__Actinomycetales;f__Geodermatophilaceae;g__Geodermatophilus;NA |
| New.ReferenceOTU942 | conventional | 1 | 0.5556 | 0.745 | 0.033 | k__Bacteria;p__Actinobacteria;c__Actinobacteria;o__Actinomycetales;f__;g__;s__ |
| New.ReferenceOTU1781 | conventional | 1 | 0.5556 | 0.745 | 0.037 | k__Bacteria;p__Actinobacteria;c__Actinobacteria;o__Actinomycetales;f__Micromonosporaceae;g__;s__ |
| New.ReferenceOTU5206 | conventional | 1 | 0.5556 | 0.745 | 0.026 | k__Bacteria;p__Actinobacteria;c__Actinobacteria;o__Actinomycetales;f__Micromonosporaceae;g__;s__ |
| New.ReferenceOTU1517 | conventional | 1 | 0.5556 | 0.745 | 0.037 | k__Bacteria;p__Proteobacteria;c__Deltaproteobacteria;o__Myxococcales;f__Cystobacterineae;g__;s__ |
| New.ReferenceOTU395 | conventional | 1 | 0.5556 | 0.745 | 0.034 | k__Bacteria;p__Firmicutes;c__Bacilli;o__Bacillales;f__Bacillaceae;g__Bacillus;NA |
| New.ReferenceOTU5268 | conventional | 1 | 0.5556 | 0.745 | 0.037 | k__Bacteria;p__Actinobacteria;c__Actinobacteria;o__Actinomycetales;f__Mycobacteriaceae;g__Mycobacterium;s__ |
| New.ReferenceOTU741 | conventional | 1 | 0.5556 | 0.745 | 0.037 | k__Bacteria;p__Firmicutes;c__Bacilli;o__Bacillales;f__Bacillaceae;g__Bacillus;s__ |
| New.ReferenceOTU1831 | conventional | 1 | 0.5556 | 0.745 | 0.021 | k__Bacteria;p__Firmicutes;c__Bacilli;o__Bacillales;f__Bacillaceae;g__Bacillus;s__ |
| New.ReferenceOTU1493 | conventional | 1 | 0.5556 | 0.745 | 0.034 | k__Bacteria;p__Actinobacteria;c__Actinobacteria;o__Actinomycetales;f__Micrococcaceae;NA;NA |
| New.ReferenceOTU683 | conventional | 1 | 0.5556 | 0.745 | 0.025 | k__Bacteria;p__Actinobacteria;c__Actinobacteria;o__Actinomycetales;f__Geodermatophilaceae;NA;NA |
| New.CleanUp.ReferenceOTU2854 | conventional | 1 | 0.5556 | 0.745 | 0.043 | k__Bacteria;p__Actinobacteria;c__Actinobacteria;o__Actinomycetales;f__Micrococcaceae;g__Nesterenkonia;s__ |
| New.CleanUp.ReferenceOTU494 | conventional | 0.8302 | 0.6667 | 0.744 | 0.05 | k__Bacteria;p__Actinobacteria;c__Actinobacteria;o__Actinomycetales;f__Micrococcaceae;g__;s__ |
| 1127976 | conventional | 0.8187 | 0.6667 | 0.739 | 0.042 | k__Bacteria;p__Actinobacteria;c__Actinobacteria;o__Actinomycetales;f__Microbacteriaceae;g__Salinibacterium;s__ |
| New.ReferenceOTU3568 | conventional | 0.8149 | 0.6667 | 0.737 | 0.048 | k__Bacteria;p__Firmicutes;c__Bacilli;o__Bacillales;f__Bacillaceae;g__Bacillus;s__ |
| New.ReferenceOTU1872 | conventional | 0.6961 | 0.7778 | 0.736 | 0.048 | k__Bacteria;p__Actinobacteria;c__Actinobacteria;o__Actinomycetales;f__Geodermatophilaceae;g__Geodermatophilus;s__ |
| 4431828 | conventional | 0.9345 | 0.5556 | 0.721 | 0.045 | k__Bacteria;p__Firmicutes;c__Bacilli;o__Lactobacillales;f__Streptococcaceae;g__Streptococcus;s__ |
| New.ReferenceOTU3270 | conventional | 0.9256 | 0.5556 | 0.717 | 0.042 | k__Bacteria;p__Actinobacteria;c__Actinobacteria;o__Actinomycetales;f__Micromonosporaceae;NA;NA |
| 588492 | organic | 0.9527 | 0.625 | 0.772 | 0.009 | k__Bacteria;p__Actinobacteria;c__Thermoleophilia;o__Gaiellales;f__Gaiellaceae;g__;s__ |
| New.ReferenceOTU4405 | organic | 1 | 0.5 | 0.707 | 0.033 | Unassigned;NA;NA;NA;NA;NA;NA |
